# Temperature expression patterns of genes and their coexpression with LncRNAs revealed by RNA-Seq in non-heading Chinese cabbage

**DOI:** 10.1186/s12864-016-2625-2

**Published:** 2016-04-22

**Authors:** Xiaoming Song, Gaofeng Liu, Zhinan Huang, Weike Duan, Huawei Tan, Ying Li, Xilin Hou

**Affiliations:** State Key Laboratory of Crop Genetics and Germplasm Enhancement/Key Laboratory of Biology and Germplasm Enhancement of Horticultural Crops in East China, Ministry of Agriculture, Nanjing Agricultural University, Nanjing, 210095 China; Center of Genomics and Computational Biology, College of Life Sciences, North China University of Science and Technology, Tangshan, Hebei 063000 China

**Keywords:** Cold and heat stresses, RNA-Seq, Expression pattern, LncRNA, Coexpression network, Non-heading Chinese cabbage

## Abstract

**Background:**

Non-heading Chinese cabbage (NHCC, *Brassica rapa* ssp. *chinensis*) is an important leaf vegetable grown worldwide. However, little is known about the molecular mechanisms underlying tolerance for extreme temperature in NHCC. The limited availability of NHCC genomic information has greatly hindered functional analysis and molecular breeding.

**Results:**

Here, we conduct comprehensive analyses of cold and heat treatments in NHCC using RNA-seq. Approximately 790 million paired-end reads representing 136,189 unigenes with N50 length of 1705 bp were obtained. Totally, 14,329 differentially expressed genes (DEGs) were detected. Among which, 10 DEGs were detected in all treatments, including 7 up-regulated and 3 down-regulated. The enrichment analyses showed 25 and 33 genes were enriched under cold and heat treatments, respectively. Additionally, 10,001 LncRNAs were identified, and 9,687 belonged to novel LncRNAs. The expression of miRNAs were more than that of pri-miRNAs and LncRNAs. Furthermore, we constructed a coexpression network for LncRNAs and miRNAs. It showed 67 and 192 genes were regulated by LncRNAs under cold and heat treatments, respectively. We constructed the flowchart for identifying LncRNAs of NHCC using transcriptome. Except conducting the *de novo* transcriptome analyses, we also compared these unigenes with the Chinese cabbage proteins. We identified several most important genes, and discussed their regulatory networks and crosstalk in cold and heat stresses.

**Conclusions:**

We presented the first comprehensive characterization for NHCC crops and constructed the flowchart for identifying LncRNAs using transcriptome. Therefore, this study represents a fully characterized NHCC transcriptome, and provides a valuable resource for genetic and genomic studies under abiotic stress.

**Electronic supplementary material:**

The online version of this article (doi:10.1186/s12864-016-2625-2) contains supplementary material, which is available to authorized users.

## Background

Nowadays, frequent occurrences of abnormal weather events have been observed all over the world, such as drought and extreme temperature. These stresses seriously impact plant growth and crops production [1, 2]. Recently, several progresses have been made about the identification of stress-related genes, which potentially are able to increase the plant tolerance [3–5]. Understanding the molecular mechanism of the abiotic stresses response is important to improve tolerance using molecular techniques.

Generally, these stress signals are converted into cellular responses through two ways, including ABA-dependent and ABA-independent signaling pathways [6, 7]. For the former, ABA is accumulated under osmotic stress caused by drought. It regulates the expression of gene under osmotic stress conditions [6, 8]. The ABA-responsive element (ABRE) is the major cis-element for ABA-responsive gene expression. ABRE-binding protein (AREB) and ABRE-binding factor (ABF) control gene expression in ABA-dependent manner [1, 9–11]. The molecular studies have revealed that ABA-independent pathway is also important for stress tolerance in plants. Dehydration-responsive element binding protein 1(DREB1)/C-repeat binding factor (CBF) and DREB2 are mainly involved in cold and heat stresses, respectively [12–15]. The DREB/CBF transcription factors (TFs) could specific bind to DRE/CRT cis-elements in promoter of target genes [14, 16, 17]. Several proteins, such as ICE1, ZAT12, CAMTA3, and MYB15, have been identified as regulators of DREB1/CBF genes [12, 18, 19]. In addition, NAC and MYB/MYC also regulate abiotic stress-responsive genes expression [20, 21]. The studies have demonstrated that there are interactions between ABA signaling pathway and other signaling factors in stress responses [1, 22, 23].

Until now, a large number of transcriptome sequencing projects have been conducted in many species. Genome-wide analyses have dramatically improved the efficiency of gene identification [16]. In *Arabidopsis*, about 30 % of the transcripts were related with abiotic stresses, and 2,409 genes played important roles in cold, salt, and drought stresses [24]. In *chrysanthemum*, 8,558 dehydration-responsive transcripts were detected using RNA-seq [25]. In wheat, about 2 % of the wheat genes were related with the cold stress [26]. In *Populus* and switchgrass, heat responsive genes were also identified by transcriptome sequencing [27, 28]. In *A. mongolicus*, 9,309 up-regulated and 23,419 down-regulated genes were identified under cold stress [29].

*Brassica rapa* contains several subspecies, such as Chinese cabbage (*B. rapa* ssp. *pekinensis*), NHCC, and turnip (*B. rapa* ssp. *rapa*) [30, 31]. The genome of Chinese cabbage had been sequenced, however, there is little information about the NHCC genome and gene dataset. Therefore, we conducted *de novo* assembly and gene annotation without prior genome information in this study. NHCC is one of the most important vegetables in China, and now is cultivated extensively worldwide. It is inevitable injured by low or heat stresses, which can directly lead to the production decrease and affect edible quality. The heat stress can affect the photosynthesis, and even induce the occurrence of several diseases, such as downy mildew, soft rot and virus diseases. The physiological change of temperature response mediated by several genes has been reported in model plants [32, 33]. However, little is known about the temperature-regulated genes and the related pathways in NHCC.

In this study, we conducted the comprehensive characterization for NHCC using RNA-seq, and explored the effect of low and heat temperature on global change. We identified several most important genes in temperature response, and discussed their regulatory networks and crosstalk in cold and heat stresses. Using Illumina sequencing technology, we generated over 85 billion base of high quality sequence, and identified a larger number of differentially and specifically expressed transcripts. Furthermore, we also identified lots of LncRNAs, and constructed the coexpression network of LncRNAs and protein encoding genes using this transcriptome dataset.

## Results and discussion

### RNA sequencing and *de novo* assembly of NHCC transcriptome

**Fig. 1:**
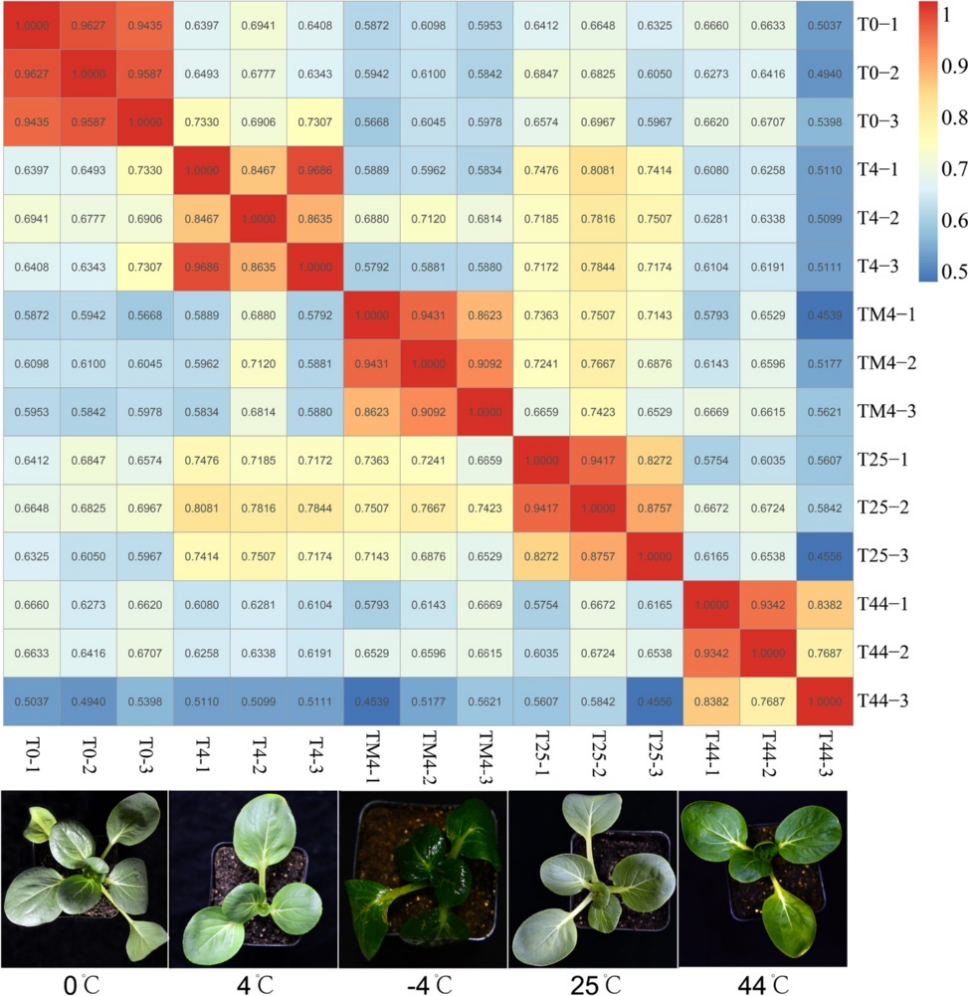
Pearson correlation coefficient analysis of all 15 libraries. The PCCs were calculated using Log2(FPKM), and the values in grid represent the PCC of any two among 15 libraries. The dashed green boxes represent the PCCs of three duplications

To obtain a global overview of NHCC transcriptome under different temperature treatments, we constructed and sequenced 15 RNA-Seq libraries, including cold treatments (4, 0 and -4 °C), heat treatment (44 °C), and normal condition (25 °C). For each temperature, three samples as the biological replications were sequenced using Illumina HiSeq™ 2000. The base quality of reads was checked using FastQC (Additional file 1: Figure S1). We used relatively stringent criteria for quality control by removing the reads with adaptors and the low quality. Finally, 790,269,418 clean pair-end (PE) reads consisting of 71.12 billion nucleotides (nt) were obtained with an average GC content of 47.30 % (Table 1, Additional file 2: Table S1). After the first assembly, 1,596,012 contigs were obtained for all libraries, and the total length over 542.8 Mb (Table 1). The contigs were further joined into136,189 unigenes using paired-end information and gap filling process. The total length of all unigenes was 153.1 Mb, and the mean length of unigene was 1124 bp (Table 1, Additional file 2: Table S2). The PE sequencing not only increases the depth, but also improves *de novo* assembly efficiency. The N50 achieved 1705 bp, which was larger than most plants *de novo* assembled by RNA-Seq, such as radish (1095 bp), wax gourd (1132 bp), and celery (1088 bp) [34–36]. This phenomenon indicated that the high quality and accuracy of our assembled transcripts. Based on FRKM, we measured the correlation of three repeats for each temperature. The results showed that there was a good correlation among three repeats. The pearson’s correlations of almost all comparisons were larger than 85 % (Fig. 1, Additional file 1: Figure S2).

**Table 1:** The summary of the sequencing and assembly

Samples	NHCC
Total raw reads	857,423,614
Total clean reads	790,269,418
Total clean nucleotides (nt)	71,124,247,620
Q20 percentage	98.05 %
N percentage	0.00 %
GC percentage	47.30 %
Contig	
Total number	1,596,012
Total length (nt)	542,865,388
Mean length (nt)	343
N50	593
Unigene	
Total number	136,189
Total length (nt)	153,124,745
Mean length (nt)	1124
N50	1705
Total consensus sequences	136,189
Distinct clusters	73,514
Distinct singletons	62,675

### Functional annotation and classification of the assembled unigenes

**Fig. 2:**
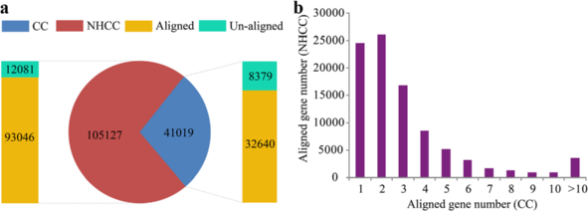
The alignment analysis for NHCC RNA-Seq transcripts and Chinese cabbage (CC) proteins. **a** The summary of the aligned and un-aligned transcripts. **b** The distribution of the aligned transcripts of the NHCC

Among all 136,189 unigenes, 121,744 (89.39 %) unigenes significantly matched a sequence in at least one of the public databases, including NCBI non-redundant protein (Nr), Gene Ontology (GO), Clusters of Orthologous Group (COG), Swiss-Prot and Kyoto Encyclopedia of Genes and Genomes (KEGG) (Additional file 2: Table S3). The size distribution of BLAST-aligned coding sequence (89.31 %) and predicted proteins are analyzed (Additional file 1: Figure S3a,b). The remaining unigenes that did not match these databases were analyzed by three programs to predict coding regions. Finally, 2793, 2491, and 3119 coding sequences were predicted by ESTScan, CPC, and CNCI programs, respectively (Additional file 1: Figure S3c, Additional file 2: Table S4). The venn diagram showed that there were 684 coding sequences predicted by these three programs, so these genes were relatively reliable as coding genes (Additional file 1: Figure S3d). A total of 105,217 coding transcripts were predicted in our study. Then we aligned these unigenes with the proteins of Chinese cabbage (E-value <10^-10^, identity >70 %). The results showed that 93,046 unigenes could align to the 3,2640 Chinese cabbage proteins (Fig. 2a). In addition, we found that over 70 % NHCC transcripts could match with more than 1 Chinese cabbage genes (Fig. 2b). This phenomenon might be caused by the genome duplication of *B. rapa*. The sequences without a homologous hit might represent novel genes in the genome, and some of them might be the specifically expressed in NHCC for temperature treatments. In addition, they also might be the non-coding, alternative transcription, lineage-specific or high allelic variant unigenes.

For Nr annotations, 104,363 unigenes matched in this database (Additional file 2: Table S3). The result indicated that 89.20 % of the top hits showed strong homology with the E-value < 1E-15 (Additional file 1: Figure S4a). The distribution pattern showed that 87.90 % of unigenes had a similarity higher than 60 % (Additional file 1: Figure S4b). The majority annotated unigenes were corresponded to the known plant genes, with 41.50 and 39.60 % matching with *A. thaliana* and *A. lyrata*, respectively (Additional file 1: Figure S4c). A total of 96,314 unigenes were assigned at least one GO term, and all GO terms were classified into three groups and further divided into 55 functional subgroups (Additional file 1: Figure S5). Overall, only 45,750 unigenes were assigned to COG classification (Additional file 2: Table S3). Among 25 COG categories, the cluster for ‘general functions prediction only’ (36.84 %) represented the largest group, followed by ‘Transcription’, and ‘Replication, recombination and repair’ (Additional file 1: Figure S6). To identify the biological pathways activated in NHCC, the assembled unigenes were annotated with KEGG. A total of 66,419 unigenes were significantly matched in this database, and were assigned to 128 KEGG pathways (Additional file 2: Table S3,5). The result showed that three largest pathway groups were metabolic pathways (ko01100, 21.12 %), biosynthesis of secondary metabolites (ko01110, 9.72 %), and plant-pathogen interactions (ko04626, 7.27 %). Following these three groups, the plant hormone signal transduction (ko04075) was about 6.60 % of all annotated genes. The level 1 of this pathway was ‘Environmental Information Processing’, and the level 2 of it was ‘Signal transduction’ in the KEGG database.

### Temperature-dependent gene expression patterns identified by RNA-Seq in NHCC

To view the gene expression, all genes were divided into three categories, including highly (FPKM >50), medium (5 < FPKM ≤50), and lowly (FPKM ≤5) expressed in each library. The results showed that most genes belonged to lowly expressed, followed by medium, and highly expressed (Additional file 1: Figure S7). The purple line shows the cumulative expressed gene number as the library number increased, and 134,980 genes were detected by all libraries.

**Fig. 3:**
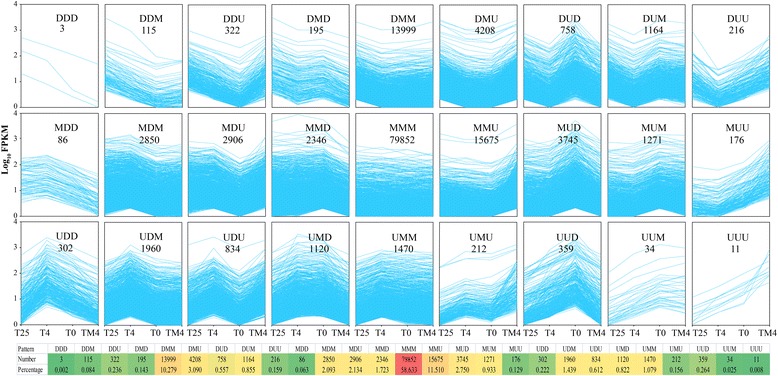
Cold stress dependent patterns of NHCC gene expression. DGEs were determined based on a combination of q-value >0.8 and FC > =2 (or < =0.5), the two sequential temperatures were compared, with the higher temperature used as the denominator. Genes were grouped into U (Up, FC > =2), D (Down, FC < =0.5), or M (Maintain, 0.5 < FC <2). Shown here are the 27 possible expression patterns. The x axis represents the four point during temperature decrease and the y axis represents the Log10 FPKM. The number shown in each box was derived based on the number of genes for each expression pattern

To evaluate the temperature decrease course and temperature-dependent transcriptomic activities during cold-resistance process in NHCC, we performed a temperature decrease course differential gene expression analysis by comparing any two adjacent cold treatments, using the higher temperature treatment as the denominator. There were 27 (3^3^) possible patterns, including those that increased across all treatment boundaries, termed‘up-up-up’ (UUU); those that were similar across all boundaries, termed ‘maintain-maintain-maintain’ (MMM); and those that decreased across all boundaries, termed ‘decrease-decrease-decrease’ (DDD). The results showed that genes were non-randomly represented across all patterns, and the overall temperature-dependent patterns are analyzed (Fig. 3, Additional file 2: Table S6). Only few genes continuously decreased (DDD) or increased (UUU) in expression accompanying temperature decrease, and the number were 3 (0.002 %) and 11 (0.008 %), respectively. However, majority genes (79,852, 58.63 %) belonged to the MMM pattern, and the expression almost unchanged over the temperature decrease process. In addition to MMM, the MMU (15,675) and DMM (13,999) were also contained more genes than other expression patterns.

### Differential expressed genes detection and compare them among each treatment

**Fig. 4:**
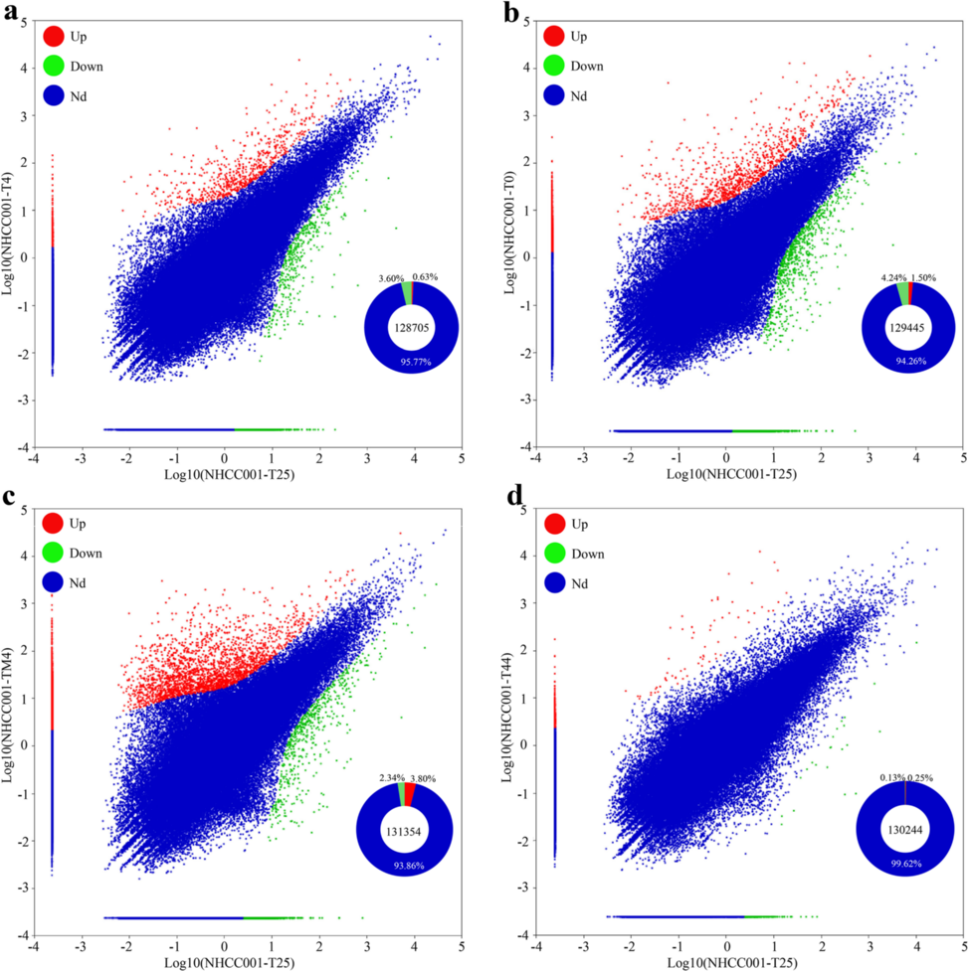
Scatter plot indicating the comparative results of log transformed gene expression levels (Log10 FPKM) and DEGs (q-value >0.8 and FC > =2 or < =0.5) distributions between control (x axis) and treatment (x axis) samples. The red dot represents up-regulated gene; the green dot represents down-regulated gene. T25, T4, T0, TM4, and T44 represent 25, 4, 0, -4, and 44 °C, respectively. **a** T25 vs T4; (**b**) T25 vs T0; (**c**) T25 vs TM4; (**d**) T25 vs T44

**Fig. 5:**
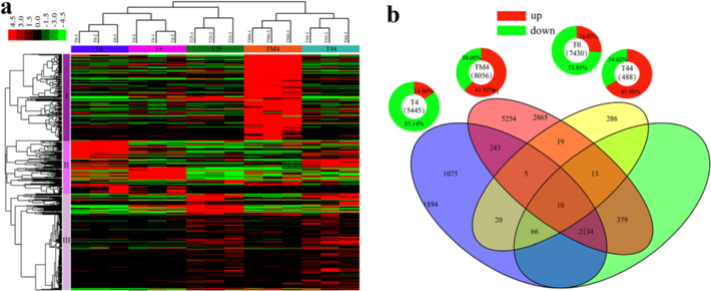
Landscape of DEGs for NHCC RNA-Seq transcriptome. **a** Hierarchical clustering analysis of gene expression profiles from 15 libraries with 14,329 DEGs. **b** The venn diagram showed the overlapping and treatment-specific DGEs in four treatments

To identify the temperature respond genes, 14,329 DEGs were detected between each temperature-treated and control library (FC >2 and q-value >0.8) (Fig. 4). All DEGs were used for clustering analysis, and obtained a well cluster results (Fig. 5a). Three repeats for each temperature got together, and 0, 4 and 25 °C formed one group, while -4 and 44 °C formed another group. On the whole, all DEGs were divided into three groups, and defined as I, II, and III. Most DEGs in group I belonged to up-regulated genes under -4 °C treatment, while similar in other four temperatures. In group II, the expression of most DEGs under 25 and -4 °C was lower than that under other three temperatures. In group III, most DEGs had relatively low expression, except a few genes under 44 °C.

To further survey the interaction of these treatments for DEGs, we constructed venn diagram using DEGs of each treatment. There were 5445, 7430, 8056, and 488 DEGs under 4, 0, -4, and 44 °C, respectively. Among of them, 1075, 2865, 5254, and 286 belonged to each treatment-specific DEGs (Fig. 5b). Interestingly, we found 10 DEGs were detected in all treatments, including 7 up-regulated and 3 down-regulated genes (Additional file 1: Figure S8). The functional annotation showed that most of them belonged to the stresses related protein, such as LEA14 and KIN2 (Table 2). In addition, we also conducted qRT-PCR experiment to verify the accuracy of the RNA-Seq. The results showed that the expression trends of all genes were consistent with the RNA-Seq, and most genes were also significant differently expressed (*p*-value < 0.01) (Additional file 2: Table S7, Additional file 1: Figure S9). Among all DEGs, 809 were up-regulated, and 1,743 were down-regulated under 4 °C; 1,937 were up-regulated, 5,493 were down-regulated under 0 °C; 4,988 were up-regulated, 3,068 were down-regulated under -4 °C. However, there were fewer DEGs under 44 °C than cold treatment, with only 322 were up-regulated and 166 were down-regulated genes (Additional file 1: Figure S10). Among these DEGs, the most treatment-specific up-regulated genes (4804) were detected under -4 °C, and the most treatment-specific down-regulated genes (1377) were detected under 0 °C (Additional file 1: Figure S8).

**Table 2:** The expression and functional annotation of 10 DEGs identified by all the cold and heat treatments. The up/down-regulated genes were identified by comparing the treatment (T4, T0, TM4, T44) and control (T25)

GeneID	T25	T4	T0	TM4	T44	Regulation	Annotation
CL4489.Contig2	0.02	24.17	22.42	26.90	11.41	Up	Unknown protein
CL10212.Contig2	0.00	6.52	4.30	7.03	4.30	Up	Glycosyl transferase family 1 protein
CL3727.Contig8	153.31	6.14	26.06	2.08	2.71	Down	Hypothetical protein ARALYDRAFT_910104
CL11270.Contig1	0.78	21.25	81.51	13.92	43.86	Up	At1g01470,a late embryogenesis abundant protein LEA14
Unigene519	0.12	209.31	1750.16	67.05	157.31	Up	BN28b, stress-induced protein KIN2 mRNA
CL8814.Contig1	139.12	12.75	2.07	1.84	3.27	Down	BN28a gene
Unigene16735	0.00	5.36	2.14	8.70	3.29	Up	Unknown protein
CL536.Contig12	0.00	3.12	7.09	10.66	8.15	Up	Wound-induced protein 1
Unigene50726	11.94	0.00	0.00	0.12	0.00	Down	ATP synthase CF1 epsilon chain,DM1-3-516-R44 chloroplast
CL2980.Contig1	8.18	203.56	272.71	191.73	317.44	Up	BN28a, stress-induced protein KIN2,Rapeseed KIN1 protein

### The enrichment analyses revealed most DEGs related with cold and heat stresses

To understand the function of DEGs, we have conducted the GO enrichment analyses using all unigenes as background (Additional file 2: Table S8). Under 4 °C, several cold related GO categories were significantly enriched, such as response to desiccation, response to cold, response to temperature stimulus, and cold acclimation. The photosynthesis, light harvesting, and translation categories were the top enrichment under 0 °C. Of course, the cold related categories were also enriched at the top 10 categories. Under -4 °C, the mainly enrichment categories were secondary metabolite biosynthetic process, S-glycoside metabolic process, glycosinolate, and glucosinolate metabolic process. However, we did not detect directly cold related categories as 4 and 0 °C at the top 10 categories. This phenomenon indicated the regulatory mechanism might exit several differences between chilling (<7 °C) and freezing (<0 °C) temperatures, which was also consistent with the previous report [37]. Under 44 °C, the mainly enrichment categories were response to high light intensity, heat acclimation, photosynthesis, and response to heat. In addition, the photosynthesis was also enriched under heat stress, which indicated that all temperature stresses could affect plant photosynthesis. We also analyzed genes belonged to the GO enrichment categories. Among all treatments, the most specific genes (2713) were found under -4 °C, followed by 0 °C (1115), 4 °C (528), and 44 °C (79). In addition, 90 genes were identified under all three cold treatments, and 5 genes were detected by all the cold and heat treatments (Additional file 1: Figure S11a).

In addition, we mapped DEGs to terms in KEGG database to identify significantly enriched pathways. Among the mapped pathways, 20, 11, 22, and 5 pathways were significantly enriched (Qvalue < 0.01) under 4, 0, -4, and 44 °C treatments, respectively (Additional file 1: Figure S12, Table S9). Notably, common enrichments were observed in photosynthesis pathway, metabolic pathway, and photosynthesis-antenna proteins pathway in all treatments. This results indicated that the cold and heat stresses affected the expression of genes involved in these pathways. Most enriched pathways were also detected by the previous reports, which partly reflected the accuracy of our results [24, 27, 29]. Interestingly, we found transcripts involved in protein processing in endoplasmic reticulum pathway were significantly enriched under 44 °C treatment, while it did not enrich in cold treatment. This phenomenon indicated that this pathway might only play roles in heat resistance. We also analyzed the genes belonged to the KEGG enrichment categories. Among all treatments, the most specific genes (1052) were found under -4 °C, followed by 0 °C (461), 4 °C (160), and 44 °C (44). Forty-five genes were identified under all three cold treatments, and 2 genes were detected by all the cold and heat treatments (Additional file 1: Figure S11b). Furthermore, we also surveyed the enrichment genes identified by combing the GO and KEGG databases. The results showed that 25 and 33 genes were enriched in the two databases under cold and heat treatments, respectively (Additional file 1: Figure S11c,d). These enriched genes will greatly enhance the potential utilization in cold and heat stresses of NHCC.

### Identification of abiotic stresses related transcription factors from DEGs

Given that TFs have a major effect on the network of temperature-responsive genes, we also identified the temperature-inducible TFs. Overall, the number of Dehydrin, Chloroa_b-bind, p450, AP2, PSI_PsaH, and EF-hand was more than other TFs in three cold treatments (Additional file 1: Figure S13a,b,c,d,e,f). However, many GST were identified under -4 °C, while they were absent under 0 and 4 °C. This phenomenon indicated that GST might play roles in the cold resistance below 0 °C. Interestingly, HSP70 and HSP20 were identified under 0 and 4 °C, indicating that there was a certain inherent association between cold and heat regulation. Under 44 °C, HSP20 was significant enriched, which revealed that it played important roles in heat-resistance regulation (Additional file 1: Figure S13g,h). In all treatments, P450 and Chloroa_b-bind TFs were enriched, which indicated that cold and heat stresses had great impact on plant photosynthesis, and thus might affect crop yields. This revealed that they were related with plant photosynthesis, which was also consistent with previous reports [38, 39].

Among all treatments, the most specific TFs were identified under -4 °C (1781), followed by 0 °C (775), and 4 °C (313) in GO enrichment (Additional file 1: Figure S14a). In addition, 51 TFs were identified under all three cold treatments, and 2 genes were detected by all cold and heat treatments. Among all KEGG categories, the most specific TFs (819) were found under -4 °C, followed by 0 °C (368), and 4 °C (128) (Additional file 1: Figure S14b). Twenty-nine genes were identified under all three cold treatments, and 1 genes were detected by all cold and heat treatments. Combing GO and KEGG enrichment analyses, 17 and 31 TFs were enriched only under cold and heat treatments, respectively (Additional file 1: Figure S14c,d, Table 3, Additional file 2: Table S10).

**Table 3:** The intersection of differntially expressed transcription factors under cold stress in GO and KEGG enrichment categories

Unigene ID	Pfam ID	TF family	E-value for Pfam	T4 *vs* T25		T0 *vs* T25		TM4 *vs* T25	
				log2 ratio	Qvalue	log2 ratio	Qvalue	log2 ratio	Qvalue
CL10543.Contig2	PF00067.17	p450	8.90E-32	6.74	0.8445	6.32	0.8308	6.34	0.838
CL11270.Contig2	PF03168.8	LEA_2	2.10E-18	5.78	0.836	6.05	0.8397	3.24	0.8154
CL11755.Contig1	PF00295.12	Glyco_hydro_28	2.60E-09	-11.3	0.8584	-11.3	0.8635	-11.3	0.8281
CL13372.Contig2	PF00657.17	Lipase_GDSL	5.20E-28	-3.71	0.8189	-3.64	0.818	-2.64	0.806
CL3153.Contig1	PF00201.13	UDPGT	3.90E-26	3.32	0.82	3.33	0.8193	2.79	0.8159
CL6375.Contig2	PF00067.17	p450	1.90E-22	-3.79	0.8254	-2.98	0.8155	-4.06	0.8365
Unigene12001	PF00764.14	Arginosuc_synth	6.50E-52	-11.6	0.8841	-11.6	0.8882	-11.6	0.8574
Unigene16263	PF00206.15	Lyase_1	1.40E-29	-11.32	0.8599	-11.32	0.865	-11.32	0.8298
Unigene20049	PF00314.12	Thaumatin	1.50E-16	-4.95	0.8083	-3.92	0.8048	-3.35	0.8017
Unigene20728	PF01676.13	Metalloenzyme	6.40E-21	-11.15	0.8443	-11.15	0.8502	-11.15	0.8126
Unigene22237	PF00504.16	Chloroa_b-bind	2.00E-48	2.49	0.8025	3.69	0.8241	3.23	0.8285
Unigene2424	PF00067.17	p450	6.80E-07	6.18	0.806	6.67	0.8301	7.13	0.8527
Unigene2446	PF00206.15	Lyase_1	3.00E-26	-11.94	0.9086	-11.94	0.9119	-11.94	0.8861
Unigene24606	PF02800.15	Gp_dh_C	4.60E-51	-12.68	0.9492	-12.68	0.9515	-12.68	0.9354
Unigene26264	PF02775.16	TPP_enzyme_C	1.10E-10	-11.31	0.8594	-11.31	0.8645	-11.31	0.8292
Unigene42146	PF00006.20	ATP-synt_ab	2.30E-33	-11.43	0.8698	-11.43	0.8745	-11.43	0.841
Unigene50595	PF00764.14	Arginosuc_synth	2.60E-26	-11.15	0.8443	-11.15	0.8502	-11.15	0.8126

**Fig. 6:**
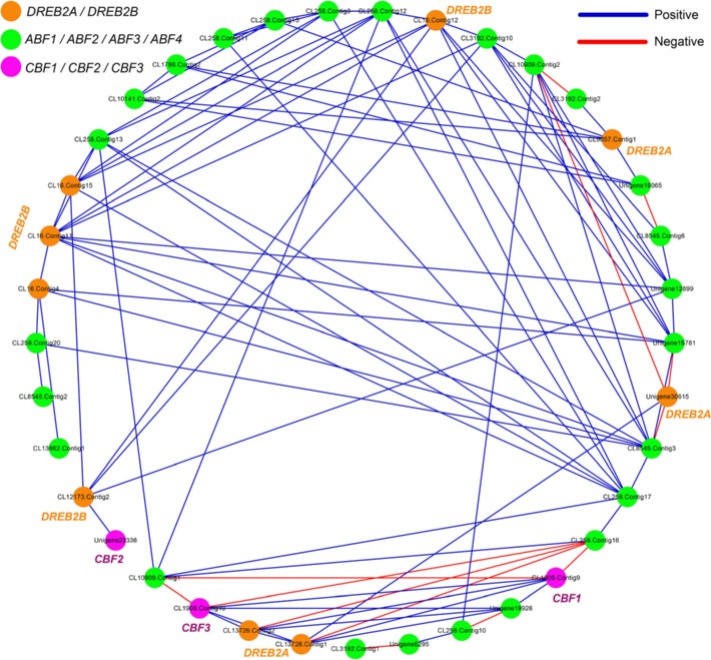
The interaction network for DREB2, CBF, and ABF TFs, which was constructed based on PCCs. The expression value of TFs at each treatment was used for calculating the PCCs. The blue lines represent the positive correlation, while the red lines represent the negative correlation

AP2/ERF TFs mainly contained two subgroups, including CBF and DREB2. They interacted with DRE/CRT cis-element and regulated ABA-independent gene expression. The CBF controlled many gene expression under several stresses, such as drought, salinity and freezing stresses. The DREB2 mainly affected gene expression under osmotic and heat stresses, while it slightly played role in cold stress [1]. Although the functions of these TFs were well explained, the interaction among them was rarely reported, especially in *B. rapa*. Therefore, we conducted correlation analysis for these TFs, and constructed the interaction network of them using expression values. A total of 38 unigenes were detected using BLAST alignment with ABFs, CBFs, and DREB2 of *Arabidopsis* (Additional file 2: Table S11). The pearson correlations coefficient (PCC) between two of these TFs were calculated using the expression value. Then the interaction network was constructed using part connections with the PCC larger than 80 %. Finally, this network contained 95 connections, including 82 positive and 13 negative connections (Fig. 6). This phenomenon revealed that most connections belonged to positive relationship among of these TFs. However, we noted that CL258.Contig16, a ABF TF, had negative connections with two DREB2A (CL13726.Contig1, CL13726.Contig2), CBF1 (CL1909.Contig9), and CBF3 (CL1909.Contig10).

**Fig. 7:**
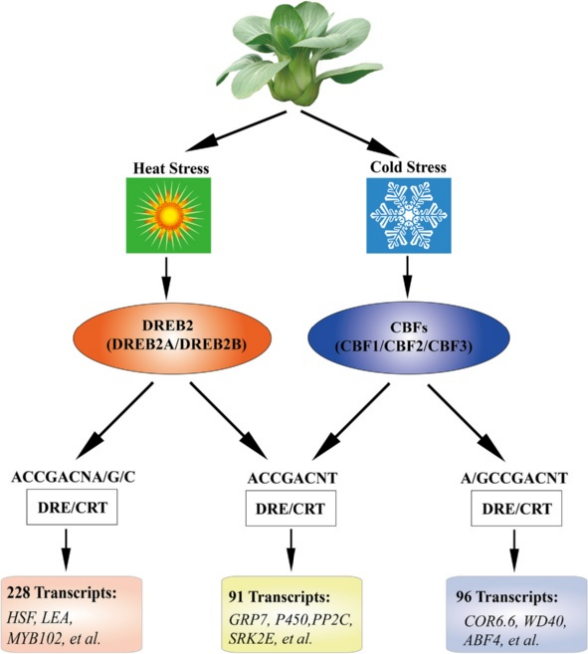
Model for DREB2 and CBF signal regulation in response to heat and cold stresses. The overlapping or specific abiotic stresses related genes were detected, which had high PCC (>90 %) with DREB2 or CBFs

To analyze the correlation of abiotic stresses and these TFs, we collected mainly cold and heat stresses related genes according to previous reports [1, 12]. Then the candidate heat and cold related genes in NHCC were identified using BLAST alignment with the collected genes. We calculated PCC of these candidate genes and CBF or DREB2. The PCC values larger than 0.8 were selected to construct interaction network (Additional file 1: Figure S15). This network showed that there were more negative connections in DREB2 than that in CBF, which only contained four negative connections. Among all connections, we identified 228 transcripts, which had high PCC (>90 %) with DREB2, such as HSF, LEA, and MYB102. By CBFs, 96 transcripts were also identified, such as COR6.6, WD40, and ABF4. Moreover, 91 transcripts were detected by both DREB2 and CBF, including GRP7, P450, PP2C, and SRK2E (Fig. 7, Additional file 2: Table S12).

### Identification and characterisation of NHCC LncRNA using RNA-seq

To identify potential LncRNA in NHCC, all sequences from NHCC transcriptome dataset were used. Based on previous reports [40–42], we designed the pipeline for LncRNA analyses. Finally, 10,001 LncRNAs were identified after a series filtering, including transcript length, coding potential, ORF size, and the exclusion of other ncRNA. Among these LncRNAs, 9,687 belonged to the novel LncRNAs. In addition, we also identified 50 pri-miRNA through comparing with Rfam and miRBase databases for comparative analyses.

### LncRNA gene expression analyses and differently expressed LncRNAs identification

**Fig. 8:**
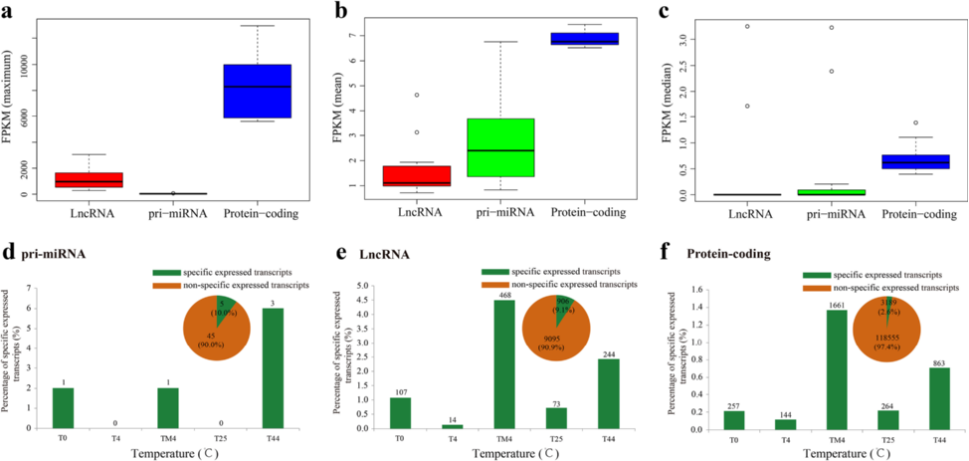
The comparative analyses of LncRNA, pri-miRNA, and protein-coding genes expression pattern. **a**, **b**, **c** The average, maximum, and median expression analysis of LncRNA, pri-miRNA, and protein coding transcripts in NHCC using boxplot. **d**, **e**, **f** The temperature specific expressed transcripts of LncRNA, pri-miRNA, and protein coding transcripts in NHCC

LncRNA is a group of endogenous RNAs that function as regulators of gene expression, which are involved in developmental and physiological processes [43]. They are longer than 200 bp, and several of them can also act as primary transcripts for the production of short RNAs [44]. We assessed the expression pattern under different temperatures using the expressed LncRNA (FPKM >0). A total of 2,236 LncRNAs were expressed in all the treatments. We observed that 73, 14, 107, 468, and 244 LncRNAs belonged to the temperature specificity for T25, T4, T0, TM4, and T44, respectively (Additional file 1: Figure S16). However, a hierarchical clustering of samples showed most LncRNA was low expression in each temperature (Additional file 1: Figure S17). In this study, 50 pri-miRNA, 10,001 LncRNAs, and 121,744 protein coding transcripts were identified. The average, maximum, and median expression values of these three type transcripts were calculated for comparative analyses. The results showed that the expression of protein coding transcripts were more than that of pri-miRNA and LncRNA (Fig. 8a,b,c, Additional file 2: Table S13). Most pri-miRNA and LncRNA had relatively low expression, which was consistent with the previous reports [45–47], indicating it was a common property of LncRNA. We also investigated the temperature specific expressed transcripts (SETs) among these three types transcripts. The results showed that 10.0 % pri-miRNA, 9.1 % LncRNA, and 2.6 % protein coding transcripts were detected as SETs. For pri-miRNA, 3 SETs were detected in 44 °C, while no SET was found in 4 and 25 °C (Fig. 8d). For LncRNA, the most SETs were identified in -4 °C (468), followed by 44 °C (244), and 0 °C (107) (Fig. 8e). The similar trends were also found in the protein-coding transcripts, except the inverse of 0 and 25 °C (Fig. 8f).

To further investigate the share or specific of these treatments for differently expressed LncRNAs (DELs), we conducted the venn diagram analyses. The results showed that 91, 418, 441, and 34 specifically expressed LncRNAs were identified under 4, 0, -4, and 44 °C, respectively (Additional file 1: Figure S18a). However, we not detected the share LncRNAs among all these treatments. Among of these DELs, most of them were down-regulated, and there were the most down-regulated LncRNAs under 0 °C (1103) than other three treatments (9 ~ 934) (Additional file 1: Figure S18b). Interestingly, the DEL number under three cold stresses (964 ~ 1327) was more than that of 44 °C, which only contained 44 DELs. However, most LncRNAs (79.55 %) were up-regulated under 44 °C, while most LncRNAs belonged to the down-regulated under other three cold treatments (56.21 % ~96.89 %).

### Construct the coexpression network between LncRNAs and protein-coding genes

We constructed a coexpression network for LncRNAs and protein-coding genes according to the previously proposed method [40]. For each treatments and for each pair of genes (LncRNA or protein-coding), we computed the PCCs of expression patterns using the expression values. We found that approximately 57.59 % were positive connections, and 42.40 % were negative connections (Additional file 1: Figure S19a). Among all connections, the PCCs of 29.27 % were between -0.4 to -0.2, and followed by 26.29 % connections were between 0.8 to 1. Furthermore, we identified 65,568,352 connections between protein-coding and protein-coding genes, among which, 42.11 % were positive connections, and 57.89 % were negative connections (Additional file 1: Figure S19b). However, the opposite result was found between LncRNA and LncRNA connections, and the values were 50.12 and 49.88 % for positive and negative connections, respectively.

To be more accurate and intuitive showed the relationship between the LncRNAs and protein-coding genes, we selected the connections with the high correlation (|PCC| > 0.95) to construct the interaction network. Overall, the whole network constituted by these connections was divided into 8 clusters, including 3 large networks and 5 relative small networks (Additional file 1: Figure S20a). In the cluster 1, about 67 % connections with the PCC > 0.95, and ~33 % with the PCC = 1. The PCCs of all connections in the cluster 2 were larger than 0.95, but less than 1. Only three connections with PCC less than -0.95 were located in the cluster1 and cluster3. Most connections (362,213) in the networks belonged to positive, and only 636 connections were negative correlation (Additional file 1: Figure S20b). This phenomenon was also found in LncRNA *vs* LncRNA and protein-coding *vs* protein-coding genes. Among all of these three types, 99.72 % connections were positive, and 0.28 % were negative connections.

To further analyze the correlation between LncRNA and protein-coding genes under temperature stresses, we annotated the function of the target genes. A total of 67 target genes were regulated by LncRNAs under all three cold treatments, and comparing them with *Arabidopsis* (Additional file 2: Table S14). The annotation showed that most of them belonged to the cold respond proteins, such as CBF1, COR6.6, and LEA14. Similar, we identified 192 target genes of LncRNAs under 44 °C treatment, and most of them belonged to the heat respond genes, such as HSP, LTP, and CBF4 (Additional file 2: Table S15). Furthermore, we also found several target genes of LncRNAs under cold and heat stresses, such as KIN2. This phenomenon indicated that they might play important roles in the corsstalk between cold and heat stresses responses.

## Conclusion

In this study, approximately 790 million paired-end reads representing 136,189 unigenes with a total length of 153.1 Mb were obtained. Only few genes were DDD or UUU expression patterns, and majority genes belonged to MMM pattern during the temperature decrease process. 14,329 DEGs were detected between at least one treatment and control library. Among which, 10 DEGs were identified in all treatments, including 7 up-regulated and 3 down-regulated genes. The enrichment analyses demonstrated that most temperature related categories were discovered under cold and heat treatments. Among the enrichment categories, 25 and 33 genes were identified in both CO and KEGG databases under cold and heat treatments, respectively.

Totally, 10,001 LncRNAs were identified from NHCC transcriptome dataset, and 9,687 belonged to novel LncRNAs. The analyses indicated the expression of protein coding transcripts were higher than that of pri-miRNA and LncRNA. We constructed a coexpression network for LncRNAs and protein-coding genes. A total of 67 and 192 target genes were regulated by LncRNAs under three cold and heat treatments, respectively. Furthermore, we also identified several shared target genes of LncRNAs under cold and heat treatments, which indicated that they might play important roles in the corsstalk between cold and heat stresses.

In conclusion, we conduct comprehensive analyses for cold and heat stresses in NHCC using RNA-seq, and identified numerous differentially and specifically expressed transcripts. Many important genes and TFs response to treatment stress were detected, and their crosstalk between cold and heat stress responses was discovered. In addition, we also identified large number of LncRNAs, and constructed the coexpression network of LncRNAs and protein encoding genes. This study provides a platform for elucidating physiologic responses to low and high temperature in *B. rapa*.

## Methods

### Plant materials, growth conditions, and treatments

The NHCC advanced inbred line, ‘Suzhouqing’, was used in this study. The surface-sterilized seeds were grown in pots containing a soil: vermiculite mixture (3:1) in a controlled-environment growth chamber programmed for 16/8 h at 25/18 °C for day/night. Seedlings at the five-leaf stage were transferred to growth chambers set at -4, 0, 4 °C as cold treatments, 25 °C as control, and 44 °C as heat treatment under 4 h for RNA-Seq and qRT-PCR. Three samples of each treatment were generated from different batches of plants for three biological replicates. All leaf samples collected from control and treated plants were washed with distilled water, immediately frozen in liquid nitrogen, and stored at −80 °C for RNA extraction.

### RNA extraction for transcriptome sequencing and RT-PCR validation

The RNA was isolated from leaves using RNA kit (Tiangen, Beijing, China) according to manufacturer’s instructions. RNA samples were treated with RNase free DNase I to avoid DNA contamination. The RNA was reverse transcribed into cDNA using Prime Script RT reagent Kit (TaKaRa, Kyoto, Japan). The cDNA libraries were constructed using an mRNA-seq assay with a fragment length range of 200 bp (±25 bp). Finally, the library was sequenced for paired-end reads of 90 bp using Illumina HiSeq™ 2000 platform, which was performed by the Beijing Genomics Institute (BGI) (http://www.genomics.cn/index). For qRT-PCR, the *actin* gene (*AF111812*) was used as an internal control to normalize the expression level of the target gene. Primer 5.0 designed the specific primers according to gene sequences. The qRT-PCR assays were performed with three biological and technical replicates. Each reaction was performed in 20-μL reaction mixtures containing a diluted cDNA sample as template, SYBR Premix Ex Taq (2×) (TaKaRa, Kyoto, Japan) and gene-specific primers. qRT-PCR was performed according to our previous report [48]. The comparative Ct value method was adopted to analyze the relative gene expression. RNA expression levels relative to *actin* gene were calculated as 2^–ΔΔCT^ according to a previous analysis [48, 49].

### Data filtering and *de novo* assembly

Raw reads generated by Illumina Hiseq™ 2000 were initially processed to get clean reads through the following three steps. i) Remove reads with adaptors contamination; ii) Discard reads with ambiguous sequences “N” larger than 5 %; iii) Remove low quality reads, which contained more than 20 % Q <20 bases [50]. In addition, we used FastQC (http://www.bioinformatics.babraham.ac.uk/projects/fastqc/) to check and visualize the quality of RNA-seq reads (Additional file 1: Figure S1). After filtering, all clean reads were assembled using a *de novo* assembly software Trinity [51]. Firstly, clean reads with a certain length of overlap were combined to generated contigs. Then, the paired-end reads were realigned to contigs to obtain unigene, which could identify different contigs in the same transcript and ensure the interval among these contigs. The contigs in one transcript were assembled by Trinity and gained the sequence not being extended on either end, which defined as unigene [34]. Then, the TGICL program was used to delete redundant unigene and further assembled all unigenes to form a single set of non-redundant unigenes [52].

### Gene expression quantification and differential expression analyses

RNA-Seq reads were aligned to the assembled transcripts using TopHat pipeline with the built-in Bowtie mapping program [53]. The expression of all unigenes was estimated by calculating read density as ‘fragments per kilobase of exon per million mapped reads’ (FPKM) [54]. The DEGs (FC >2, q-value >0.8) between normal and stress-treated conditions were identified using NOISeq (http://www.bioconductor.org/) [55]. GO enrichment analyses were performed using Blast2GO [56]. The temperature-dependent gene expression patterns were analyzed according to the previous report [57]. The comparisons were made between two adjacent temperatures—that is, 25 °C *vs* 4 °C, 4 °C *vs* 0 °C, and 0 °C *vs* -4 °C. A gene with FC > =2 was grouped into ‘up’ pattern, a gene with FC < =0.5 was grouped into ‘decrease’, and the remaining genes were grouped into ‘maintain’. Therefore, a gene was grouped to 1 out of 27 patterns, ranging from up-up-up (UUU), maintain-maintain-maintain (MMM), to decrease-decrease-decrease (DDD).

### Functional annotation and classification of the transcripts

All assembled transcripts were annotated with the publicly available protein databases, including Nr (http://www.ncbi.nlm.nih.gov), GO (http://www.geneontology.org), COG (http://www.ncbi.nlm.nih.gov/COG), Swiss-Prot protein (http://www.expasy.ch/sprot), and KEGG (http://www.genome.jp/kegg) databases using BLAST (E-value <10^-5^). Then, the best alignments were used to decide sequence direction and to predict coding regions of the unigenes. ESTScan software was used to decide sequence direction and coding regions when a unigene unaligned to none of the above databases [58]. WEGO software was used to conduct GO classification for understanding the distribution of gene function [59]. The unigenes were also aligned to COG database to predict and classify possible functions. In addition, KEGG was used to annotate the pathway of the unigenes.

### LncRNA detection

To *de novo* detect LncRNAs using RNA-seq, we developed a flowchart according to previous reports with slightly modification (Additional file 1: Figure S21) [40, 41, 60, 61]. We applied several filters to ensure reliability of LncRNAs. Firstly, all unigenes (136,189) were annotated using BLAST (E-value <10^-5^) alignment with NR,NT,Swiss-Prot,KEGG,COG, and GO databases. There were 14,445 unigenes un-annotated by any protein databases above mentioned. Among which, 14,300 unigenes selected for transcripts greater than or equal to 200 bp. Secondly, Coding-Non-Coding Index (CNCI, http://www.bioinfo.org/software/cnci) was applied on all candidate unigenes in order to distinguish protein-coding and non-coding sequences [62]. The unigenes with score <0 were defined as non-coding. In addition, Coding Potential Calculator (CPC, http://cpc.cbi.pku.edu.cn) was also used for identifying all candidate transcript models in order to assess their coding potential by a second independent method [63]. In order to extract potential non-coding transcripts with a high reliability from our dataset, all transcripts with a score (CPC < -1) were retained as potential non-coding. By combining these two methods, 10,930 unigenes were identified as potential non-coding RNAs. Thirdly, we discard transcripts with an ORF greater than 100 amino acids by ORF-Predictor (E-value <10^-5^), and 813 unigenes were discard.

Finally, 10,117 candidate transcripts were identified and compared against several non-coding RNA databases, including Rfam, miRBase, NONCODE with designated threshold value (E-value <10^-5^, identity >90 %) by BLAST [64–66]. Candidate transcript models with known protein motifs were discarded. We obtained 50 pri-miRNA sequences by comparing with the miRBase and Rfam databases. To identify LncRNA, we filtered other non-coding RNAs through comparing with Rfam and NONCODE databases. Finally, 10,001 unigenes were identified as LncRNA based on a series of analyses above mentioned. Furthermore, among which, 9,687 belonged to the novel novel LncRNAs by comparing with NONCODE databases.

### Statistical analysis

The differential expression levels of genes under the different temperatures were clustered using Cluster program (http://bonsai.hgc.jp/~mdehoon/software/cluster), and visualized using Tree View software (http://jtreeview.sourceforge.net/) [67]. Using in-house Perl script, PCCs were calculated for correlation studies, including the three repeats correlation, LncRNA and target mRNA correlation, and key candidate mRNA-mRNA correlation. The coexpression interaction networks were constructed using Cytoscape (http://www.cytoscape.org/) according to PCC [68]. The numbers of specific and common genes were plotted using Venn diagram in R package [69].

### Ethics and consent to participate

This article does not contain any human or animals data performed by any of the other committee.

### Consent to publish

Not applicable.

### Availability of data and materials

The RNA sequence dataset supporting the results of this article is available in available on NHCC Data Center under Project P002 (http://nhccdata.njau.edu.cn/).

## Additional files

Additional file 1: Figures S1-S21. (PDF 2762 kb) Additional file 2: Tables S1-S15. (XLS 9790 kb)

## Abbreviations

DEGs: differentially expressed genes
DELs: differently expressed LncRNAs
FPKM: fragments per kilobase of exon per million mapped reads
NHCC: Non-heading Chinese cabbage
PCC: pearson correlations coefficient
SETs: specific expressed transcripts
