# Supplementary material for: Temperature expression patterns of genes and their coexpression with LncRNAs revealed by RNA-Seq in non-heading Chinese cabbage
Source: BMC Genomics. 2016 Apr 22;17:297. doi: 10.1186/s12864-016-2625-2 (PMC4840866; doi:10.1186/s12864-016-2625-2)
Supplement: Additional file 1: — Figures S1-S21. (PDF 2762 kb) [file 12864_2016_2625_MOESM1_ESM.pdf]

Temperature expression patterns of genes and their coexpression with LncRNAs revealed by RNA-Seq in non-heading Chinese cabbage

Xiaoming Song[1, 2], Gaofeng Liu[1], Zhinan Huang[1], Weike Duan[1], Huawei Tan[1], Ying Li[1] & Xilin Hou[1, *]

[1]State Key Laboratory of Crop Genetics and Germplasm Enhancement/Key Laboratory of Biology and Germplasm Enhancement of Horticultural Crops in East China, Ministry of Agriculture, Nanjing Agricultural University, Nanjing 210095, China.

[2]Center of Genomics and Computational Biology, College of Life Sciences, North China University of Science and Technology, Tangshan, Hebei 063000, China.

*****Corresponding author: Email: hxl@njau.edu.cn

Supplementary Figures

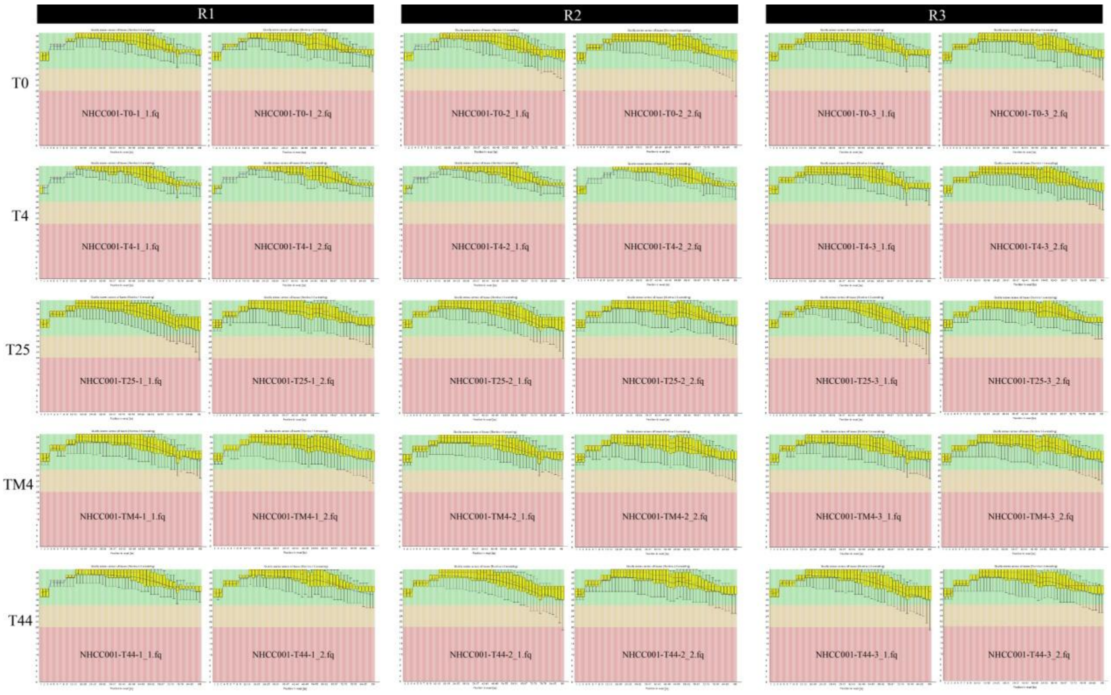

Figure S1 Quality for each base in reads viewed by software FastQC.

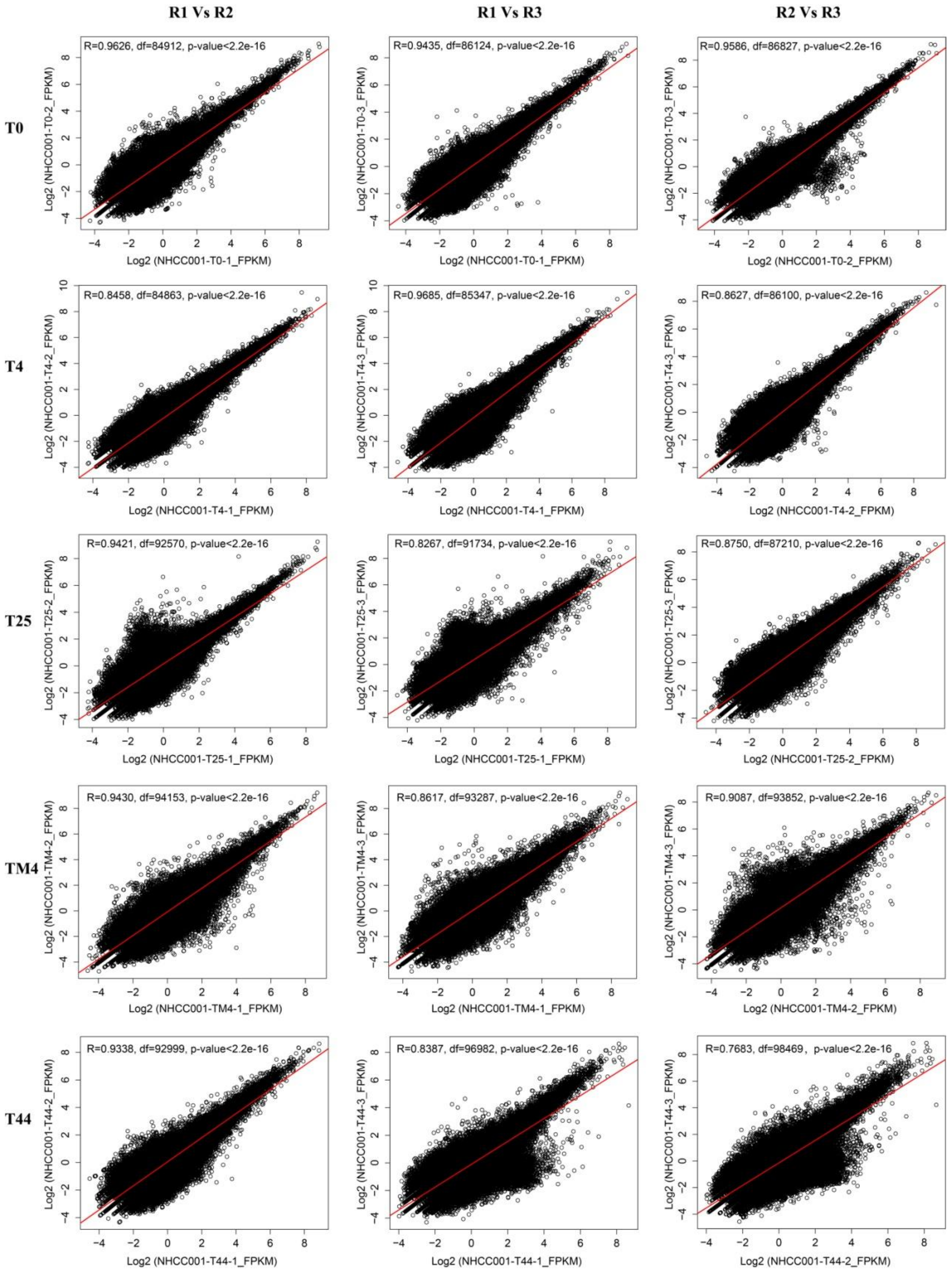

Figure S2 Comparison of gene expression in any two libraries of three repeats. The Pearson's correlation (R value) was calculated between the log2-transformed FPKM values of two libraries.

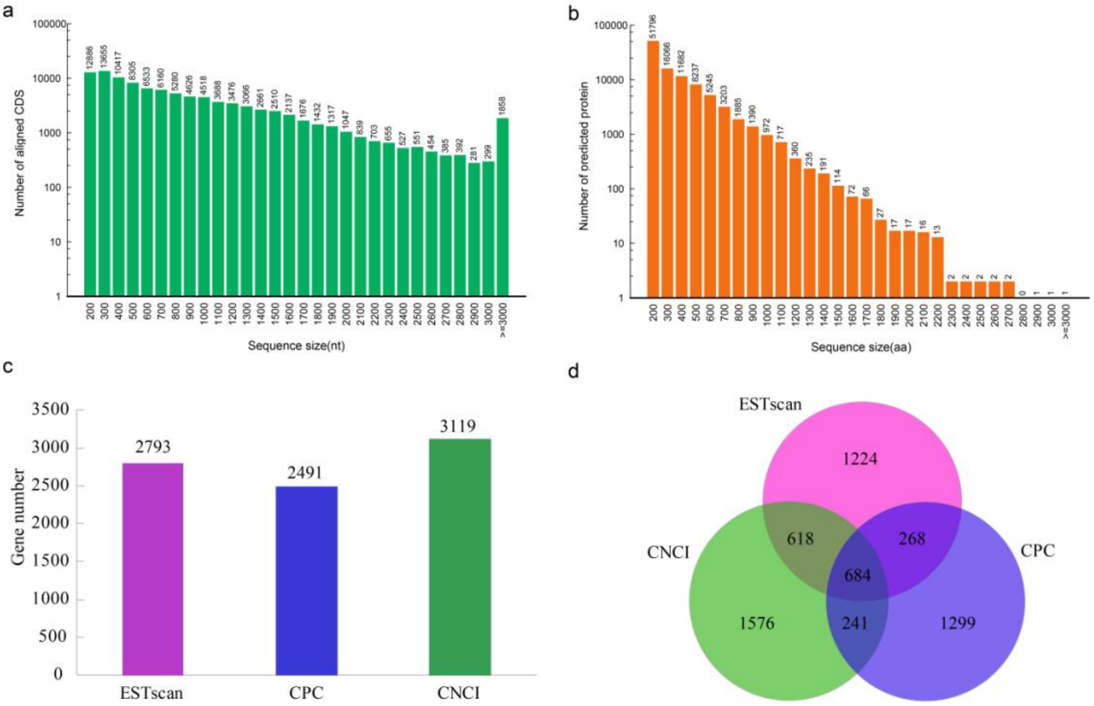

Figure S3 The analysis of the coding sequence (CDS) and predicted proteins from unigenes. (a) The length distribution of the aligned CDS by BLASTX; (b) The length distribution of the predicted proteins by BLASTX; (c) The number of CDS predicted by ESTScan, CPC, and CNCI programs; (d) The venn diagram of the predicted CDS by three programs.

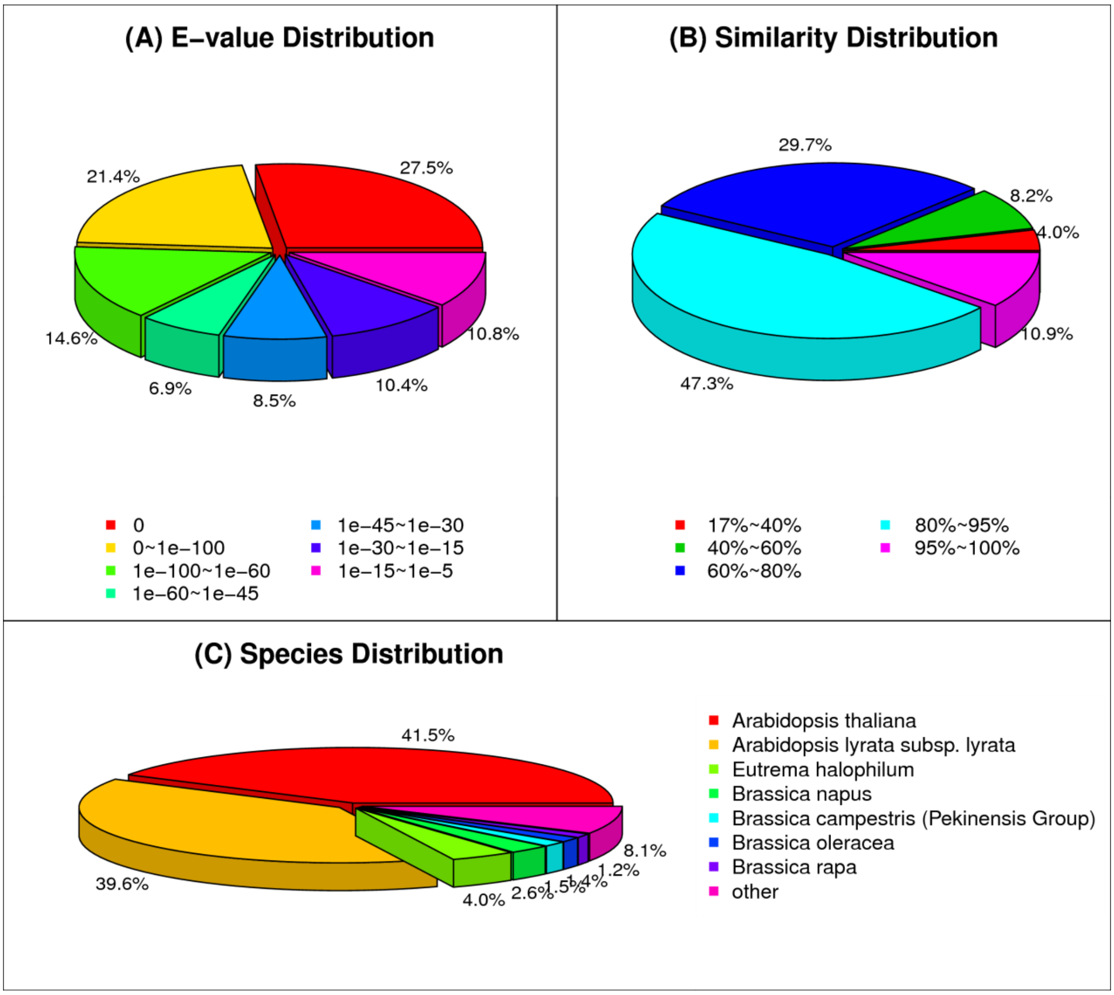

Figure S4 Characteristics of sequence homology of NHCC BLAST against NCBI non-redundant (NR) database. (a) E-value distribution of BLAST hits for matched unigenes with E-value cutoff of 1.0E-5; (b) Similarity distribution of top BLAST hits for each unigene; (c) Species distribution of the top BLAST hits.

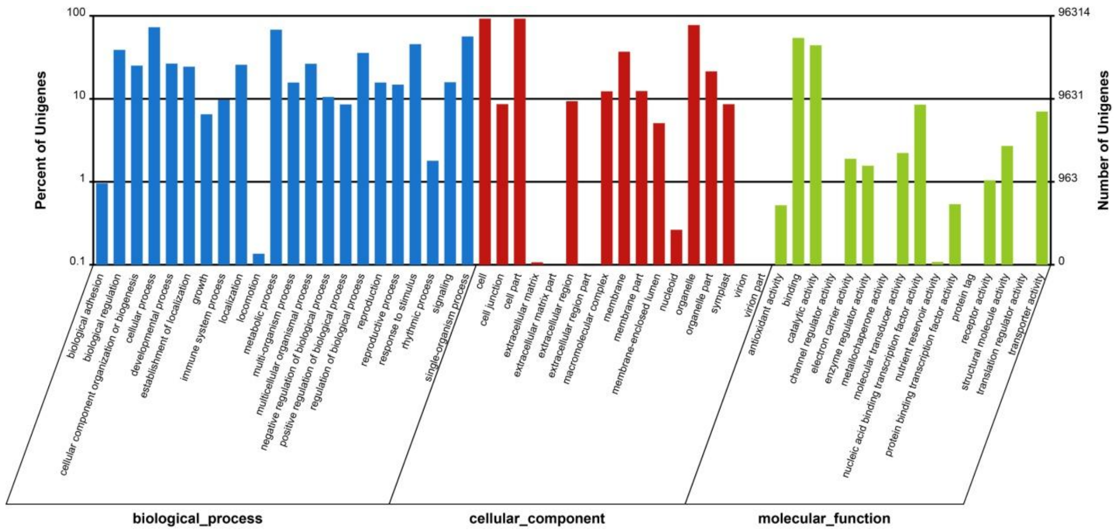

Figure S5 Gene ontology classification of the unigenes in NHCC.

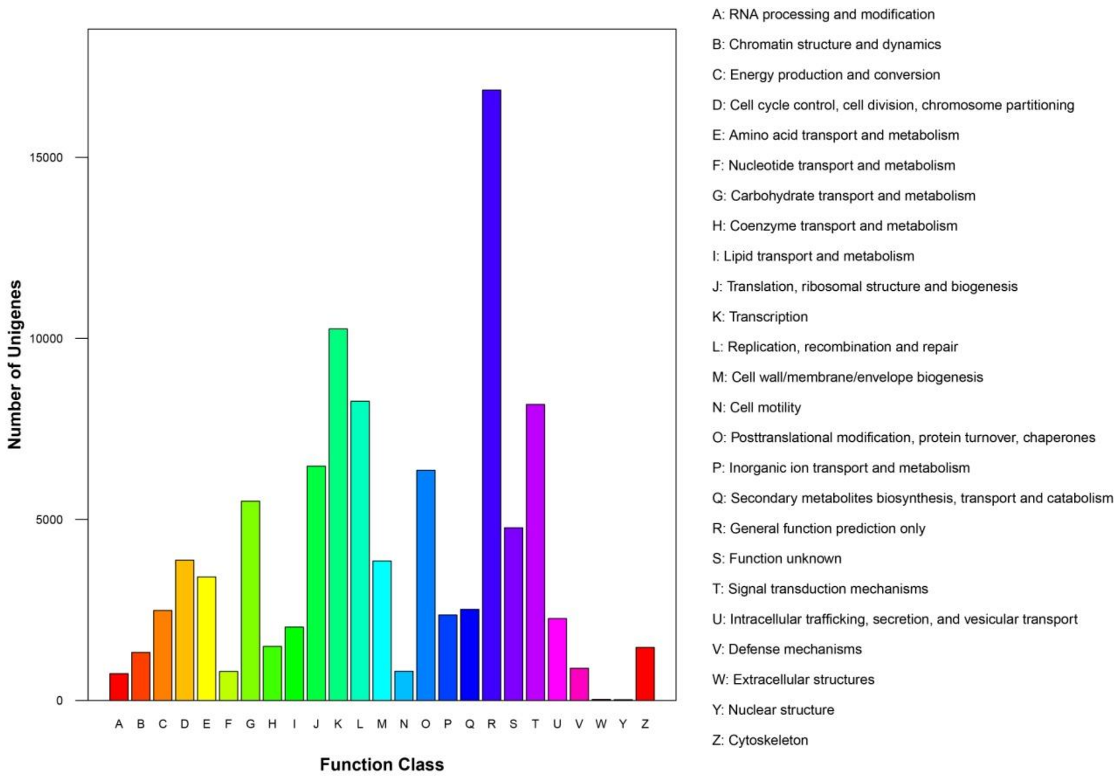

Figure S6 COG function classification of the unigenes in NHCC.

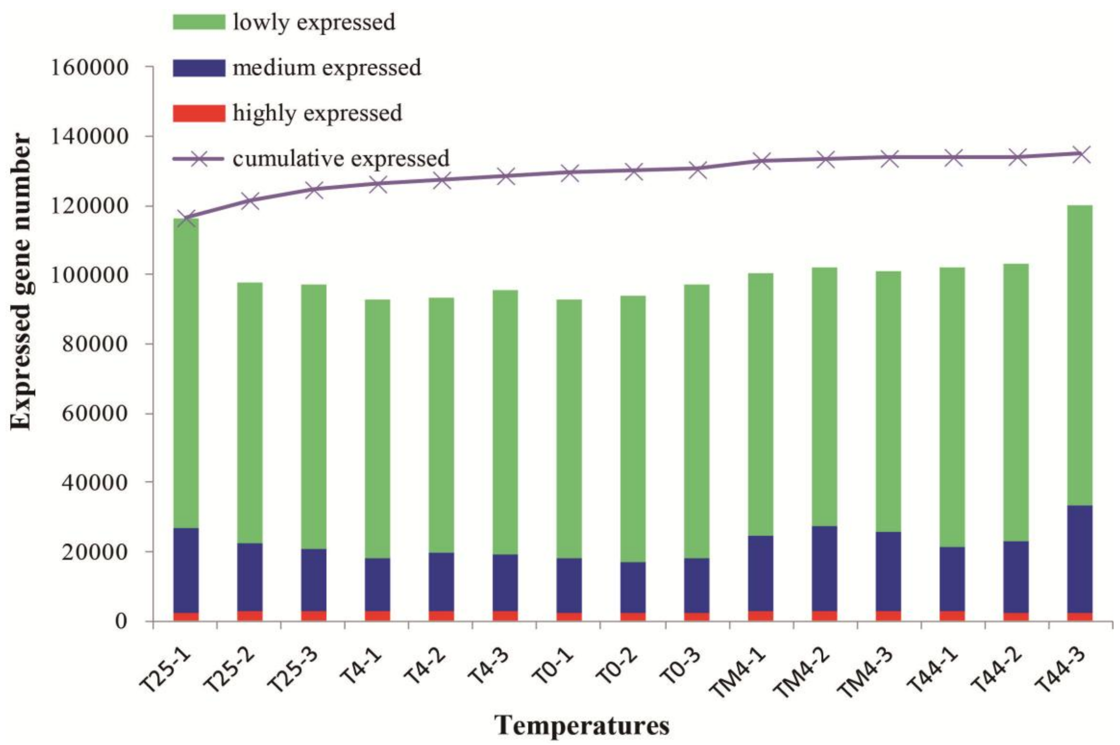

Figure S7 The number of highly (FPKM > 50), medium (5 < FPKM ≤50), and lowly (FPKM ≤ 5) expressed genes in each libraries. The purple line shows the cumulative expressed gene number as the library number increased.

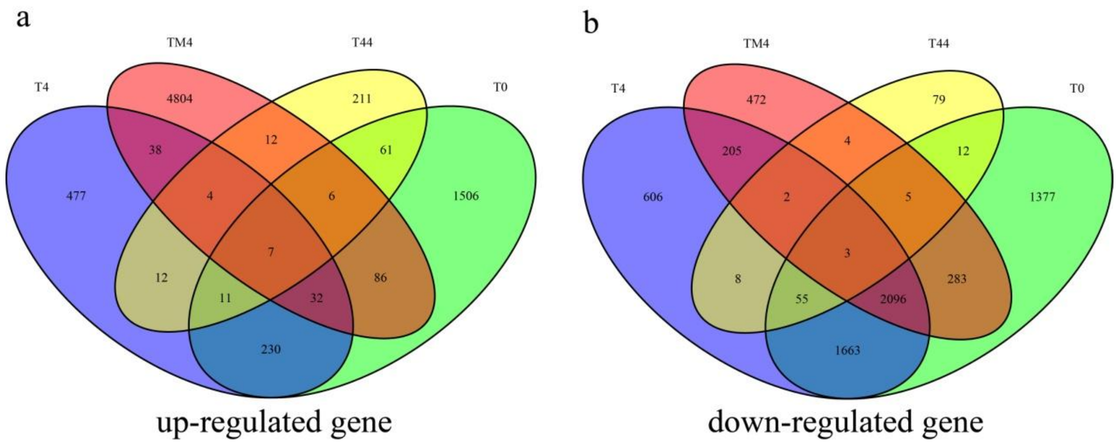

Figure S8 The venn diagram showed the overlapping and treatment-specific DGEs in four treatments. (a) up-regulated genes; (b) down-regulated genes.

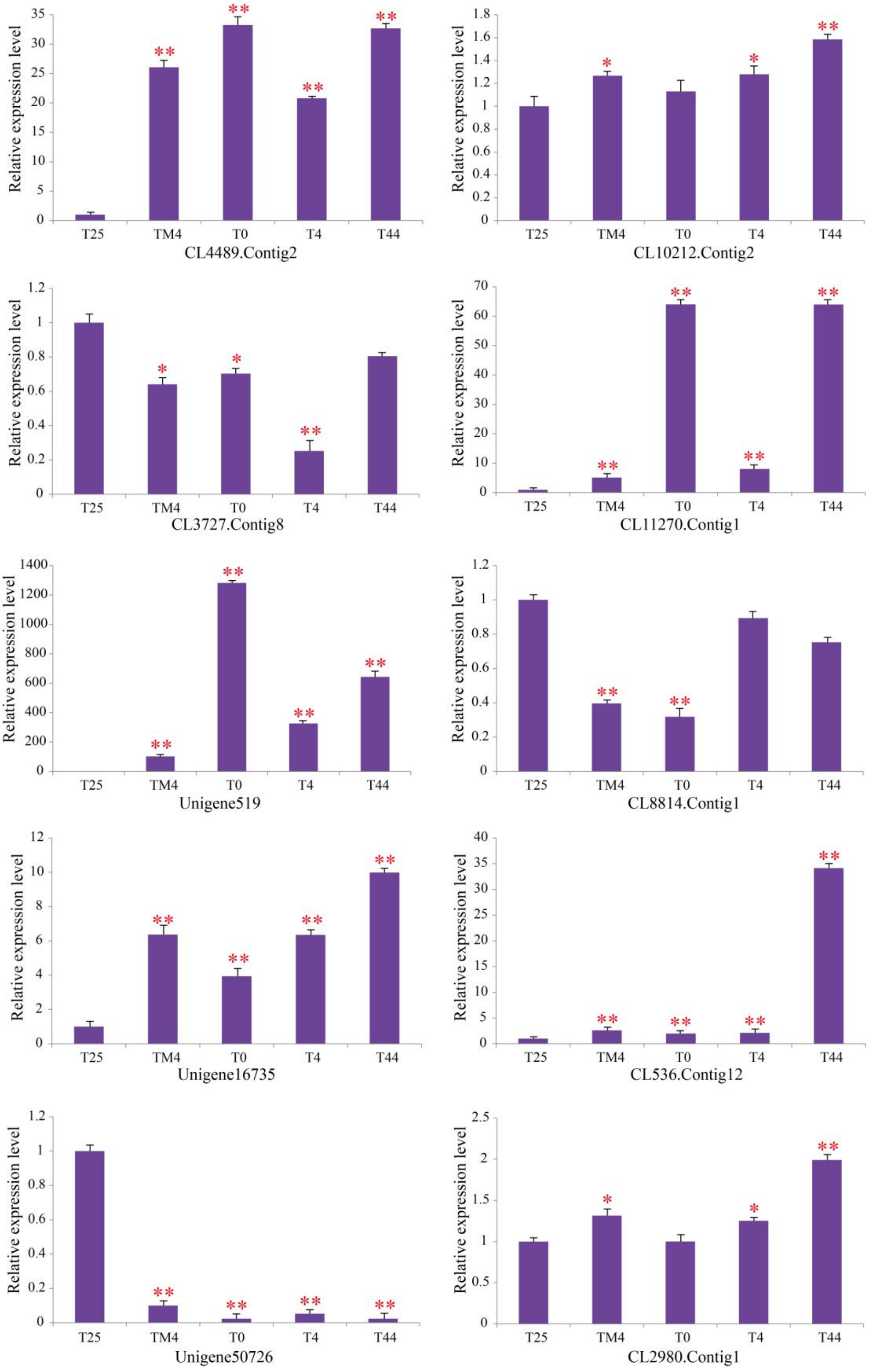

Figure S9 Quantitative RT-PCR analysis of ten DEGs under 25℃, -4℃, 0℃, 4℃, and 44℃ treatments. Metric bars represent the standard error (SE). Asterisks indicate significant differences: * $P < 0.05$, ** $P < 0.01$.

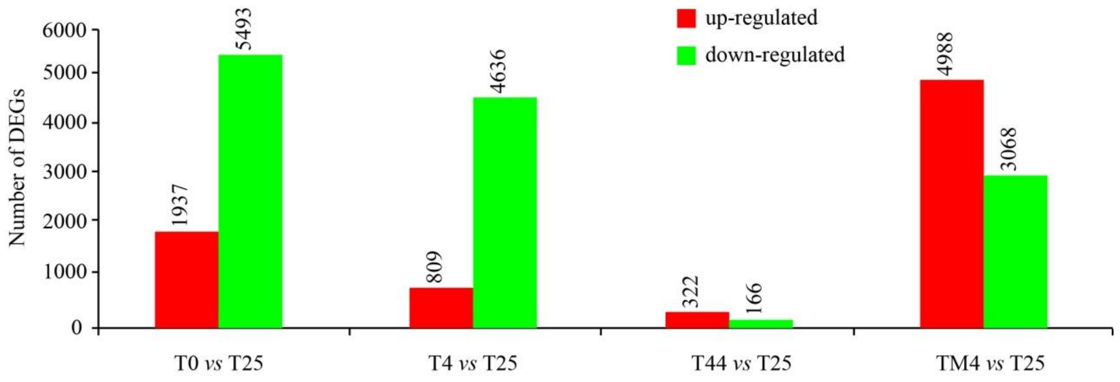

Figure S10 The number of up-regulated and down-regulated genes for each treatment.

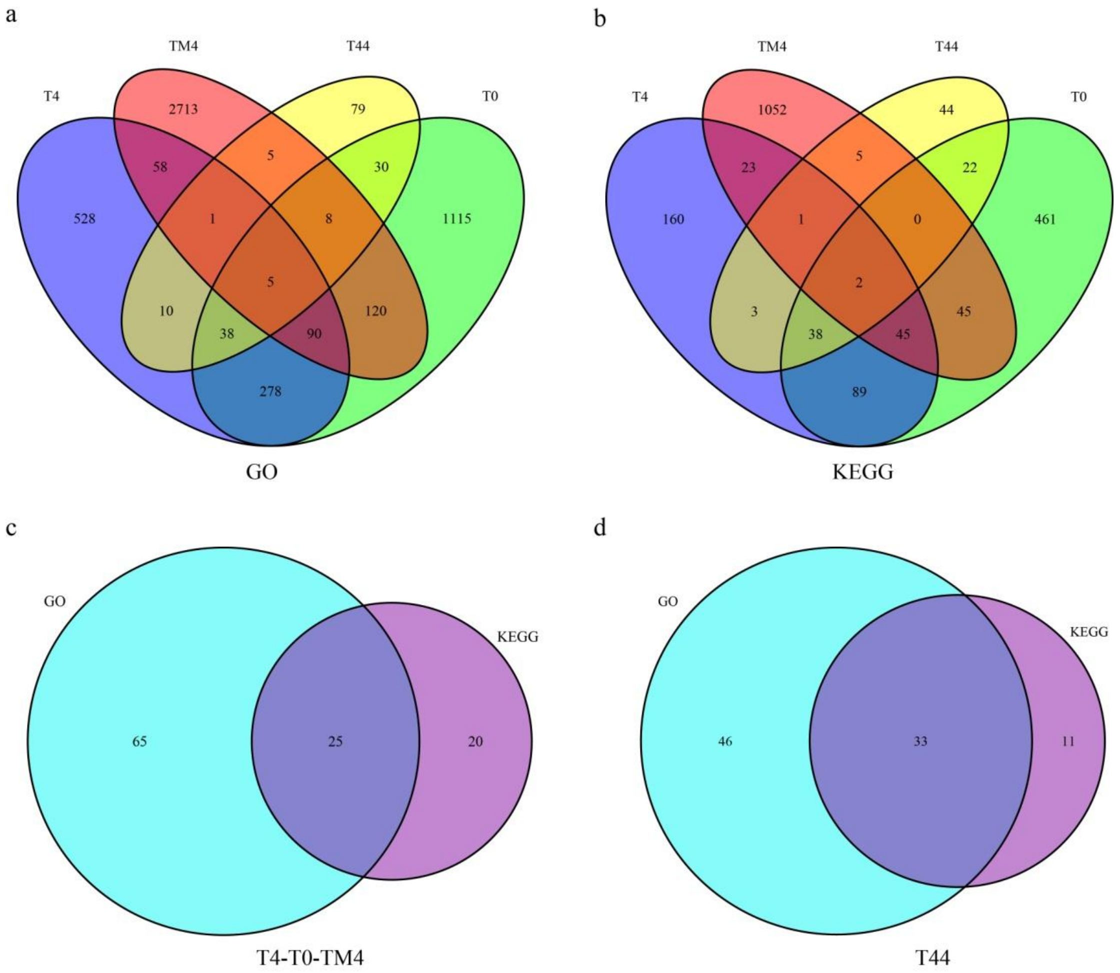

Figure S11 The enrichment DGEs analyses using GO and KEGG databases. (a) The overlapping and treatment-specific enrichment DGEs in four treatments detected by GO. **(b)** The overlapping and treatment-specific enrichment DGEs in four treatments detected by KEGG. (c) The overlapping and treatment-specific enrichment DGEs of all three cold treatments identified by combing GO and KEGG databases. (d) The overlapping and treatment-specific enrichment DGEs of heat treatment identified by combing GO and KEGG databases.

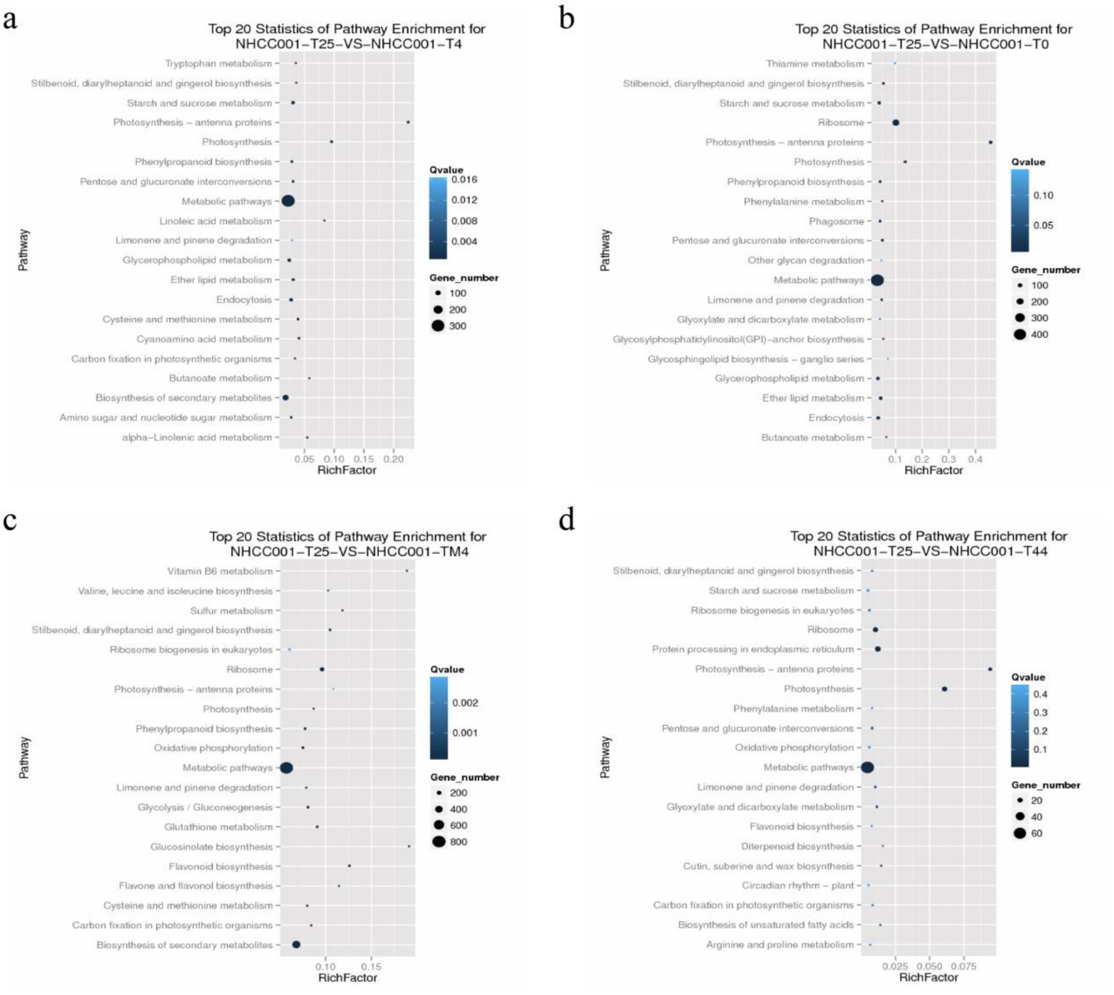

Figure S12 The significantly enriched metabolic pathways or signal transduction pathways of DEGs in KEGG database.

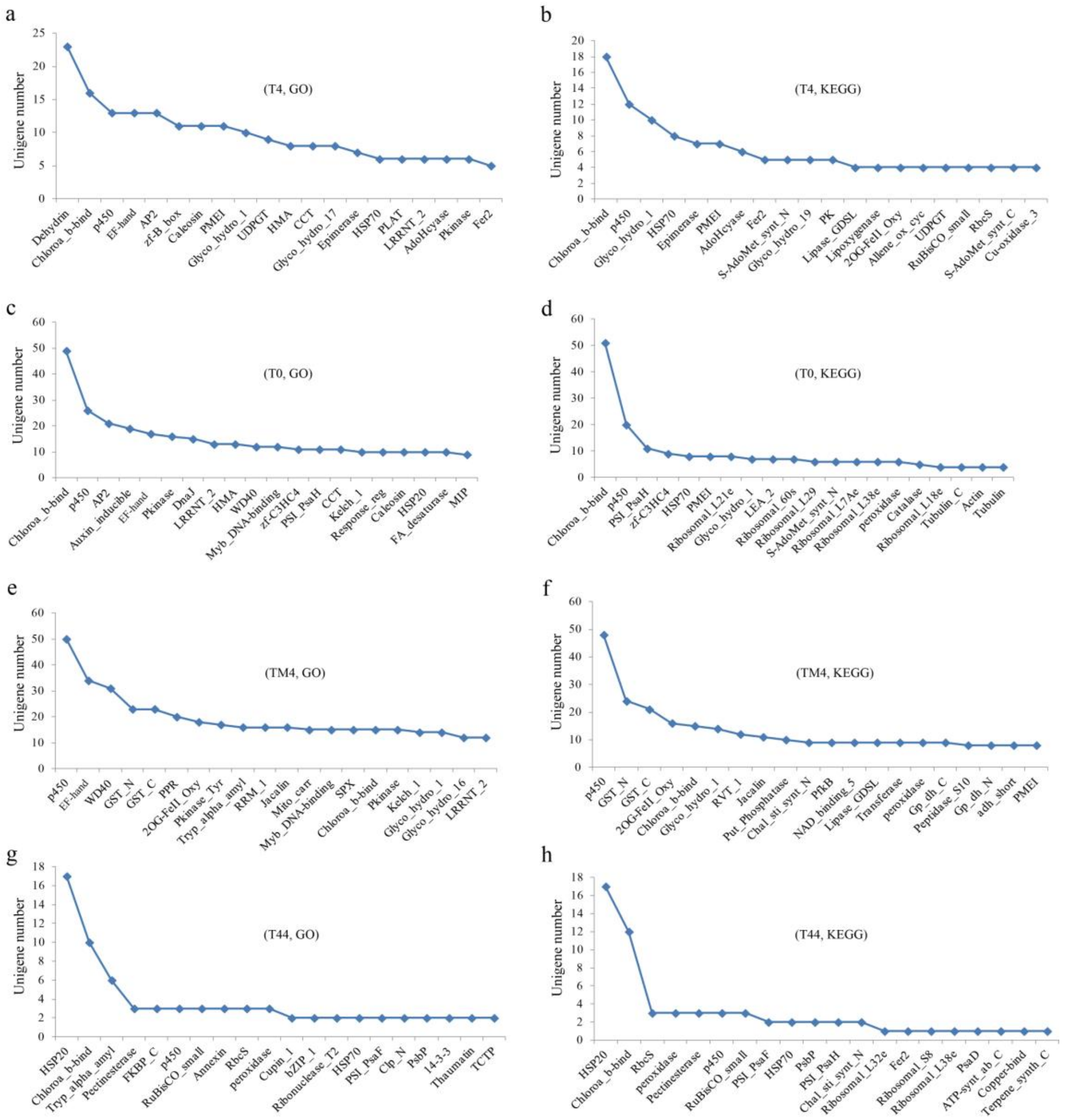

Figure S13 The TFs identified from the GO and KEGG enrichment categories for each treatment. (a,b) The TFs numbers for 4℃ treatment identified from the GO and KEGG enrichment categories, respectively. (c,d) The TFs numbers for 0℃ treatment identified from the GO and KEGG enrichment categories, respectively. (e,f) The TFs numbers for -4℃ treatment identified from the GO and KEGG enrichment categories, respectively. (g,h) The TFs numbers for 44℃ treatment identified from the GO and KEGG enrichment categories, respectively.

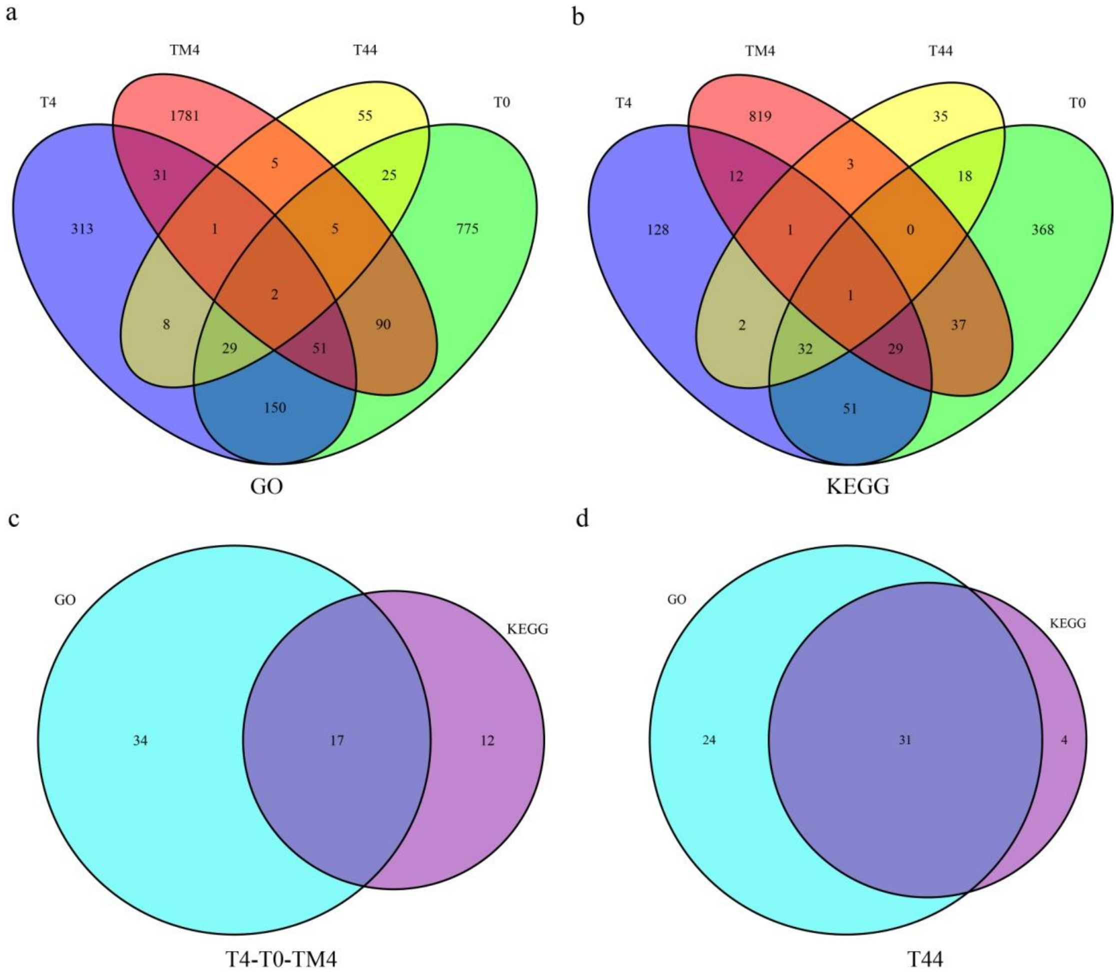

Figure S14 The TFs identified from the enrichment DGEs of GO and KEGG databases. (a) The overlapping and treatment-specific TFs in four treatments detected by GO. **(b)** The overlapping and treatment-specific TFs in four treatments detected by KEGG. (c) The overlapping and treatment-specific TFs of all three cold treatments identified by combing GO and KEGG databases. (d) The overlapping and treatment-specific TFs of heat treatment identified by combing GO and KEGG databases.

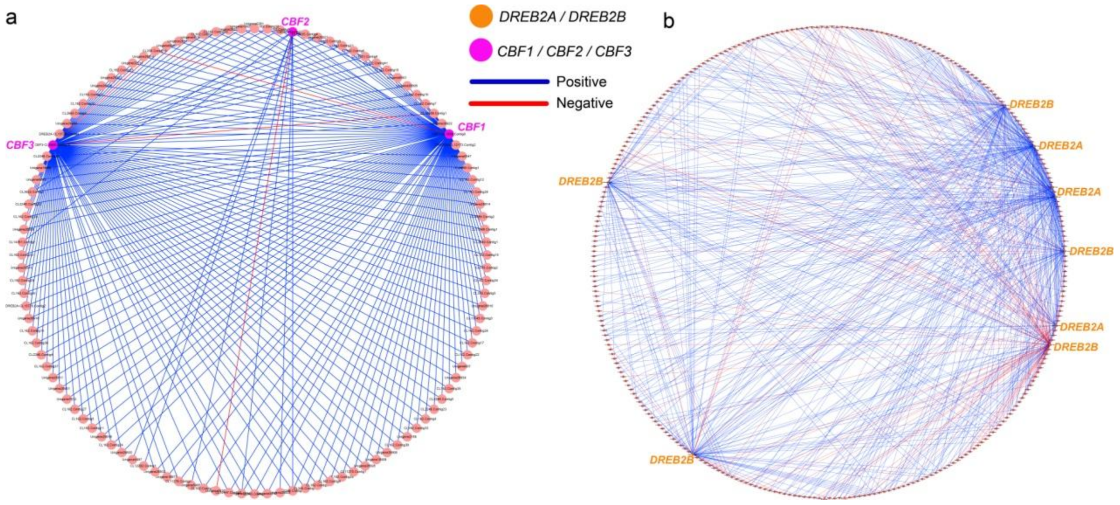

Figure S15 The interaction network of cold and heat related genes with (a) CBF or (b)DREB2.

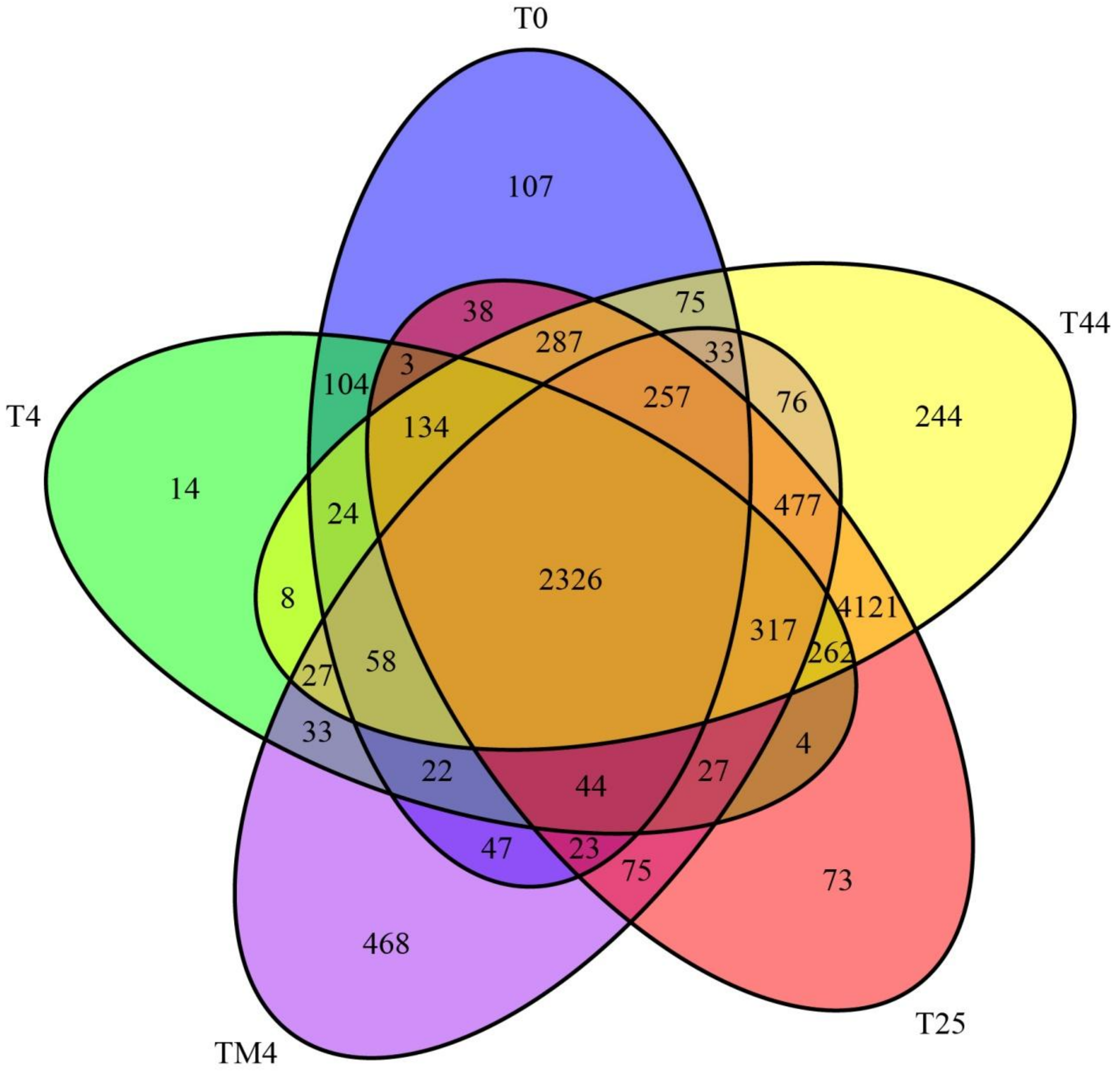

Figure S16 The venn diagram showed the overlapping and treatment-specific expressed LncRNA among five temperatures.

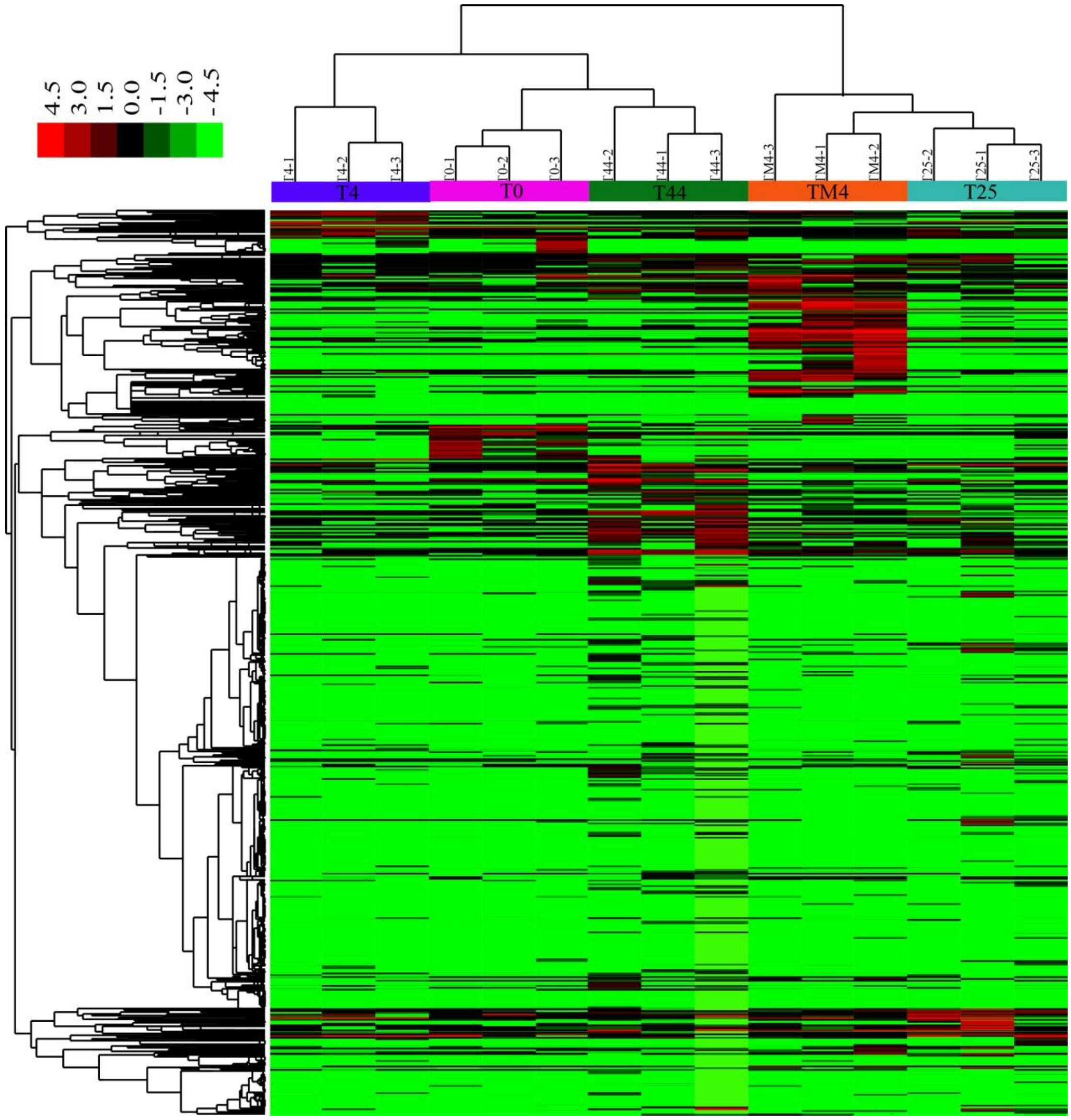

Figure S17 Hierarchical clustering analysis of LncRNA expression profiles from 15 libraries.

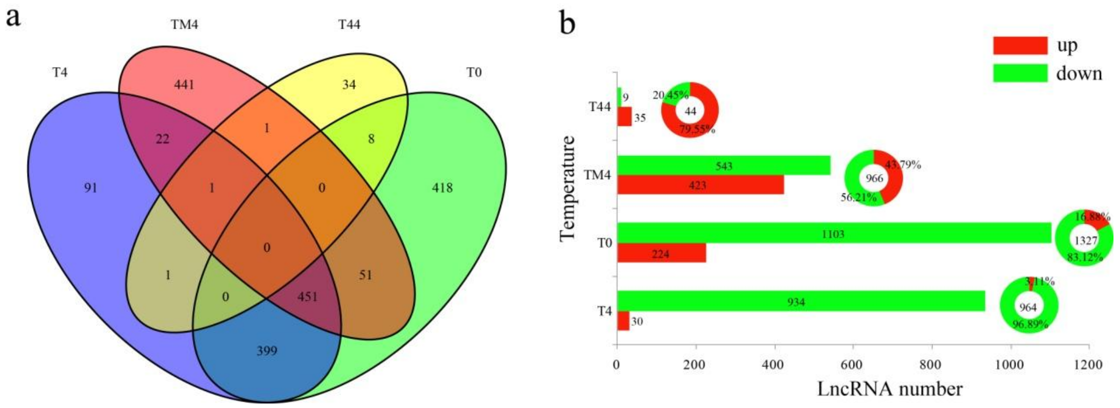

Figure S18 Landscape of differentially expressed LncRNAs (DELs) for NHCC RNA-Seq transcriptome. (a) The venn diagram showed the overlapping and treatment-specific DELs in four treatments. (b) The number of up-regulated and down-regulated LncRNAs for each treatment.

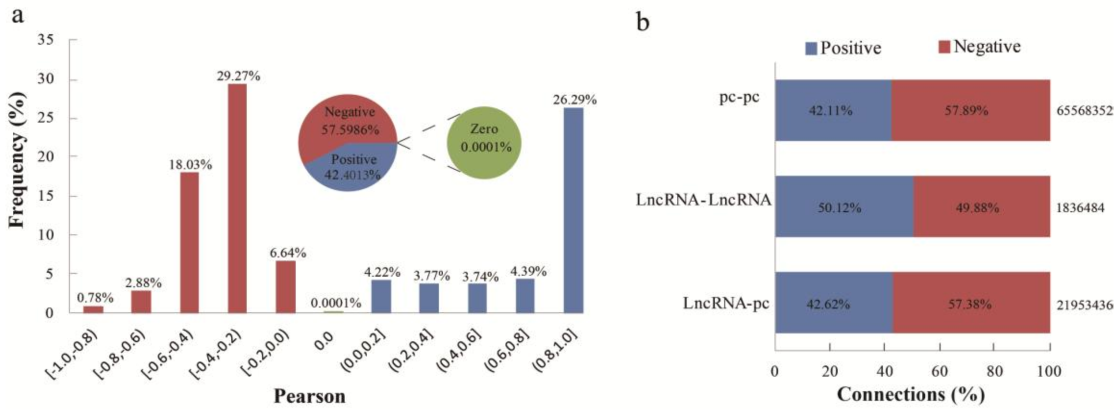

Figure S19 Characteristics of connections of LncRNAs and protein-coding genes. (a) Frequency distribution histogram of PCCs of LncRNA-mRNA expression levels across 16 libraries (b) Number of positive and negative connections for three categories.

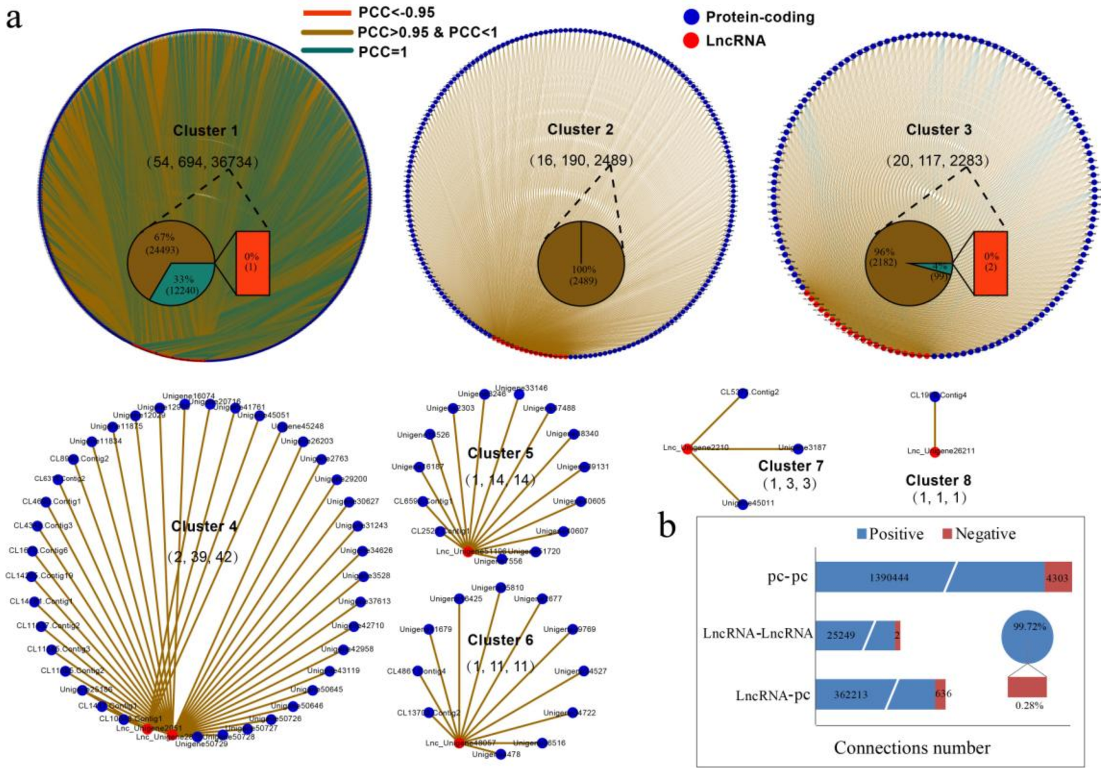

Figure S20 Co-expression network of protein-coding genes and LncRNAs with the absolute PCC >0.95. (a) Cytoscape representation of the 8 clusters in the co-expression network. The values in brackets represent the number of LncRNAs, protein-coding genes, and connections, respectively. (b) Number of positive and negative connections for three categories. pc–pc, connections between two protein-coding genes; LncRNA–LncRNA, connections between two LncRNAs; LncRNA-pc, connections between a protein-coding and a LncRNA gene.

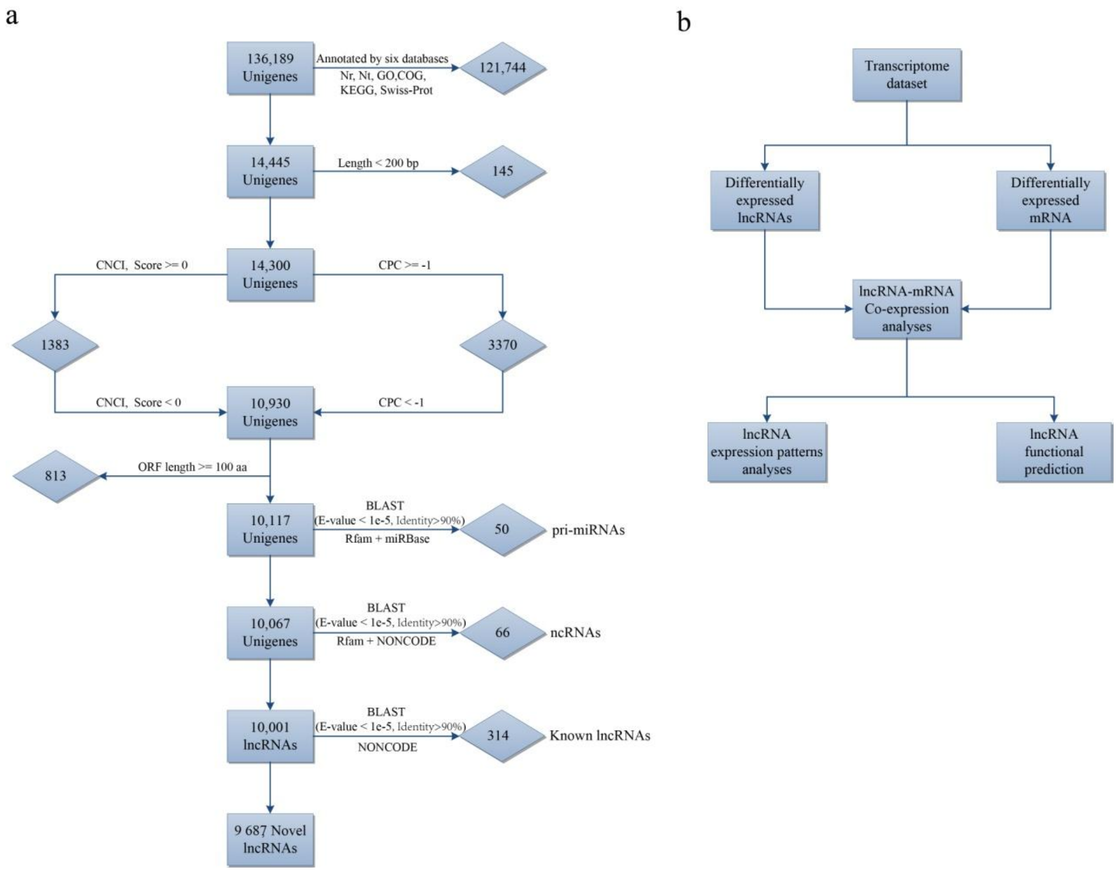

Figure S21 The flowchart for LncRNA analyses. (a) The pipeline for LncRNA identification using RNA-Seq dataset. (b) The pipeline for constructing the co-expression network between LncRNA and protein-coding genes.
